# Leveraging epigenomes and three-dimensional genome organization for interpreting regulatory variation

**DOI:** 10.1371/journal.pcbi.1011286

**Published:** 2023-07-10

**Authors:** Brittany Baur, Junha Shin, Jacob Schreiber, Shilu Zhang, Yi Zhang, Mohith Manjunath, Jun S. Song, William Stafford Noble, Sushmita Roy

**Affiliations:** 1 Wisconsin Institute for Discovery, University of Wisconsin-Madison, Madison, Wisconsin, United States of America; 2 Paul G. Allen School of Computer Science and Engineering, University of Washington, Seattle, Washington, United States of America; 3 Department of Bioengineering, University of Illinois at Urbana-Champaign, Urbana, Illinois, United States of America; 4 Carl R. Woese Institute for Genomic Biology, University of Illinois at Urbana-Champaign, Urbana, Illinois, United States of America; 5 Department of Physics, University of Illinois at Urbana-Champaign, Urbana, Illinois, United States of America; 6 Cancer Center at Illinois, University of Illinois at Urbana-Champaign, Urbana, Illinois, United States of America; 7 Department of Genome Sciences, University of Washington, Seattle, Washington, United States of America; 8 Department of Biostatistics and Medical Informatics, University of Wisconsin-Madison, Madison, Wisconsin, United States of America; Academy of Mathematics and Systems Science, Chinese Academy of Science, CHINA

## Abstract

Understanding the impact of regulatory variants on complex phenotypes is a significant challenge because the genes and pathways that are targeted by such variants and the cell type context in which regulatory variants operate are typically unknown. Cell-type-specific long-range regulatory interactions that occur between a distal regulatory sequence and a gene offer a powerful framework for examining the impact of regulatory variants on complex phenotypes. However, high-resolution maps of such long-range interactions are available only for a handful of cell types. Furthermore, identifying specific gene subnetworks or pathways that are targeted by a set of variants is a significant challenge. We have developed L-HiC-Reg, a Random Forests regression method to predict high-resolution contact counts in new cell types, and a network-based framework to identify candidate cell-type-specific gene networks targeted by a set of variants from a genome-wide association study (GWAS). We applied our approach to predict interactions in 55 Roadmap Epigenomics Mapping Consortium cell types, which we used to interpret regulatory single nucleotide polymorphisms (SNPs) in the NHGRI-EBI GWAS catalogue. Using our approach, we performed an in-depth characterization of fifteen different phenotypes including schizophrenia, coronary artery disease (CAD) and Crohn’s disease. We found differentially wired subnetworks consisting of known as well as novel gene targets of regulatory SNPs. Taken together, our compendium of interactions and the associated network-based analysis pipeline leverages long-range regulatory interactions to examine the context-specific impact of regulatory variation in complex phenotypes.

## Author summary

Single nucleotide polymorphisms (SNPs) are single base pair changes in the human genome that can contribute to variations in complex phenotypes, including diseases. Many SNPs lie in non-coding parts of the genome, far away from genes, making it difficult to identify downstream molecular pathways that are affected by these variants. Long-range gene regulation wherein a regulatory sequence element affects the expression of a gene hundreds of kilobases away, has emerged as a possible mechanism by which non-coding SNPs impact the function of a gene. Hi-C is a high-throughput genomic assay that can be used to infer three-dimensional organization and long-range interactions. However, collection of Hi-C datasets across diverse cell types and contexts is a significant challenge. In our study, we used publicly available datasets to train a model to predict Hi-C measurements across multiple cell types. We use these predictions to infer long-range interactions between SNPs and genes and combine with network-based approaches to identify potential downstream pathways disrupted by these SNPs. Our collection of long-range interactions, affected pathways and analysis approach should be a useful resource to uncover biological pathways that are affected by non-coding genomic variation for different disease and normal conditions.

## Introduction

Genome-wide association studies (GWAS) have identified a large number of variants associated with different phenotypes and diseases [1]. Approximately, 93% of all GWAS variants are regulatory variants, located in non-coding regions that can regulate gene expression, with nearly 20% located over 100kb away from any genic feature [2,3]. Understanding the mechanisms by which such variants contribute to phenotypic variation is a significant challenge because the target genes and pathways of non-coding variants as well as the specific cell types in which these variants operate are unknown. Recent studies have shown that regulatory sequences such as enhancers can harbor non-coding variants that impact gene expression [4–7] in a cell-type-specific manner [8,9]. Three-dimensional organization of the genome enables long-range regulatory interactions between distal enhancers and genes through chromosomal looping that brings the enhancer in spatial proximity to target genes. Consequently, long-range interactions are emerging as an important component for the interpretation of regulatory variation [8–10] and have been implicated in many diseases such as autoimmune diseases [10] and cancer [11]. To systematically examine the impact of regulatory variants, we need long-range regulatory interaction maps across multiple cell types as well as computational tools that can leverage these interactions to identify the gene networks that are targeted by sets of regulatory variants.

Genome-wide Chromosome Conformation Capture (3C) technologies, such as Hi-C [12], are experimental methodologies for measuring genome-wide maps of 3D proximity of genomic loci from which we can potentially determine which physical interactions have regulatory roles. However, detecting long-range interactions between regulatory elements and genes requires high-resolution Hi-C datasets (e.g., 5kb), which are limited to a handful of well-characterized model cell types due to sequencing costs and the large number of cells required for making reliable measurements at high resolution. Micro-C, which was recently developed for mammalian cell types, generates higher resolution datasets but requires more cells than Hi-C, roughly 5 million, and is only available for a small number of cell types [13]. Because of these technical challenges, a number of computational approaches have been developed to predict long-range interactions using classification [14–16] and regression approaches [17,18]. While many of these approaches have primarily used sequence [19–21], several others have additionally used one-dimensional epigenomic signals such as histone modifications, transcription factor (TF) binding and accessibility, which are widely available for many cell types. However, prediction of counts and interactions in new cell types remains difficult and requires a large number of datasets for training, which may not be available for most cell types and tissues.

A related challenge is identifying interactions that link regulatory variants to their target genes and the impact such interactions have on downstream pathways. Computational approaches to identify molecular networks that are impacted by sequence variants have largely focused on coding variants [22]. Recent computational approaches have generated cell-type-specific enhancer functional networks linking distal regions to disease [23] and enabled the identification of non-coding risk variants by leveraging disease-relevant gene regulatory networks [24]. While both approaches accurately identify disease-relevant genomic loci, neither identify the target genes or gene pathways. Another recent approach identifies cell-type-specific enhancers harboring GWAS variants, but requires measured Hi-C to link them to genes [25]. Furthermore, to our knowledge, no existing approach has examined the downstream pathways impacted by long-range regulatory interactions.

**Fig 1:**
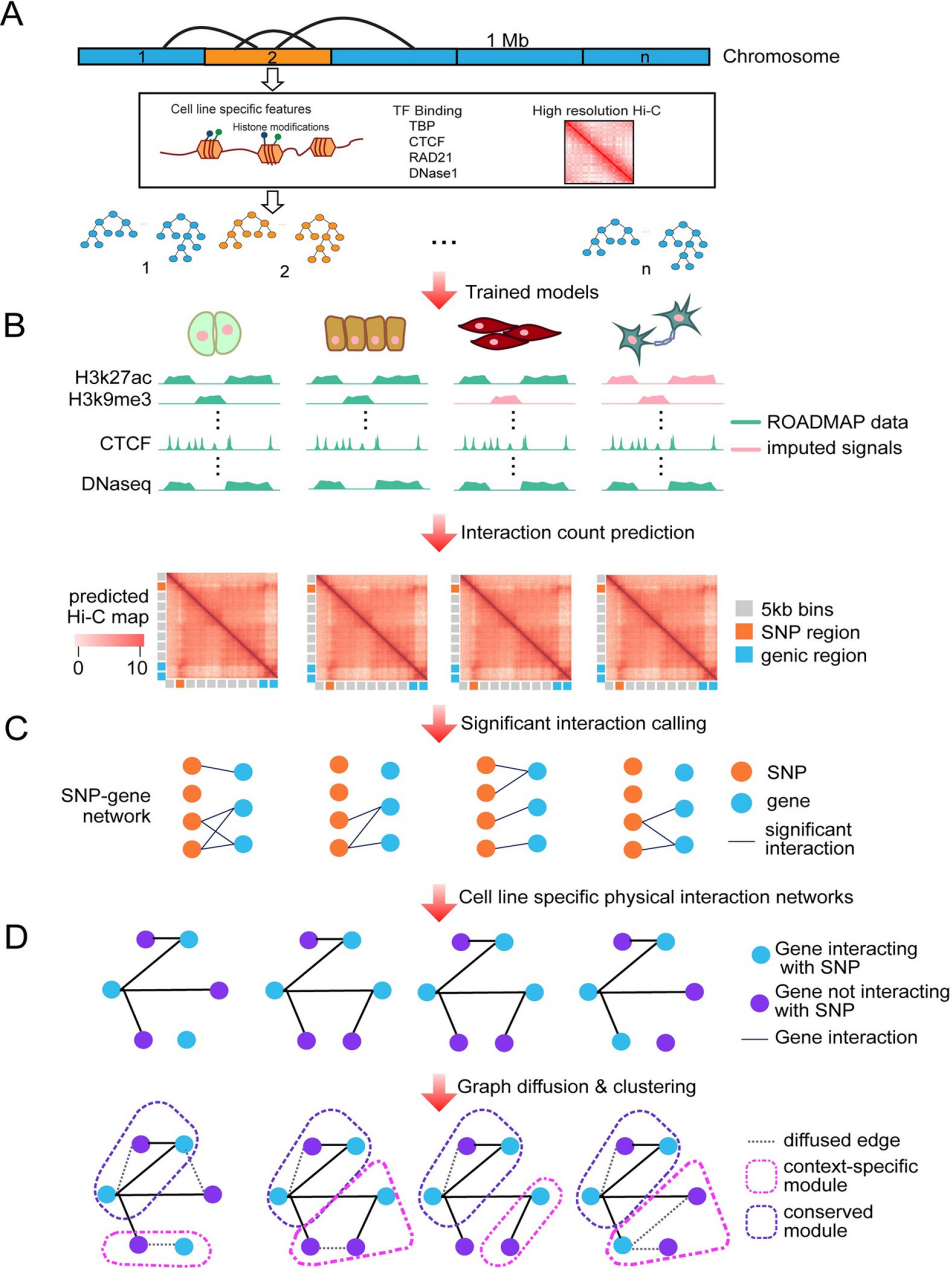
Overview of long-range interaction prediction and graph-based variant interpretation. **A.** L-HiC-Reg is trained on 1 MB regions of a chromosome with one-dimensional regulatory genomic datasets and high resolution Hi-C data using a random forest regression algorithm. **B.** The trained models are then applied to measured and imputed datasets in the Roadmap epigenomics database to generate a compendium of predictions in 55 cell types. Generic 5kb bins are shown in gray, bins overlapping SNPs are in orange and TSS bins are in blue. **C.** SNPs and genes are connected in the SNP-gene network via long-range interactions with the SNPs for a given phenotype. Genes are scored based on the average significance of interactions with SNPs **D.** Physical molecular interactions from protein-protein interaction networks and transcription factor (TF)-gene interaction networks are used to perform graph diffusion on the scores from **C.** Multi-task graph clustering is used to identify gene subnetworks jointly across each cell type to identify pathways affected by the set of SNPs.

To address both these challenges – the lack of comprehensive high-resolution cell-type-specific long-range regulatory interactions and defining the target pathways of a set of regulatory variants– we developed a computational pipeline with two components: L-HiC-Reg (Local HiC-Reg), to predict long-range interactions, and graph-diffusion coupled with multi-task graph clustering, to identify the gene networks targeted by a set of variants in a cell type-specific manner (**Fig 1**). L-HiC-Reg is a Random Forests regression method that adapts our previous tool, HiC-Reg [18], to leverage the local structure of genomic segments, improving generalizability across cell types and tissues. We used L-HiC-Reg models trained on high-resolution Hi-C data to generate a compendium of *in silico* Hi-C maps for 55 publicly available cell types and tissues using experimental and imputed data from the Roadmap epigenomics database [26]. We then leverage the compendium to link non-coding SNPs to target genes via long-range regulation. Compared to existing tools that predict enhancer-gene relationships such as GeneHancer [27] and JEME [14], our compendium is both cell-type-specific and is based on regression, rather than classification. The advantage of the regression approach is that it does not rely on specific peak calling tools to define an interaction, which may bias the results. Classification-based approaches would need to use an interaction calling tool [15,16,28] to generate training pairs, and these tools can vary in the number of interactions detected and the range of distance between pairs in detected interactions [29]. Generation of the negative set could also introduce additional biases because of the paired nature of Hi-C interactions and the lack of true negative interactions [26,27]. Furthermore, predicting counts directly allows the end user the flexibility to apply different statistical tools including peak calling and topologically associated domain (TAD) analysis. Our multi-task graph clustering approach identifies gene subnetworks for a particular phenotype of interest in a cell-type-specific manner. Previous approaches to link non-coding SNPs to target genes have focused solely on the direct target genes using predicted or measured chromatin loops [22,23,24]. By contrast, our approach integrates both distal and proximal interactions and identifies downstream pathways that may be disrupted by a set of SNPs. Our predicted long-range interactions are corroborated with experimentally derived interactions from complementary ChIA-PET and Capture-Hi-C experiments and link more sequence variants to genes compared to existing approaches.

We applied our approach to fifteen different phenotypes from the NHGRI-EBI GWAS catalog that represent different autoimmune, cardiovascular, neurological and cancer phenotypes [1]. We identified gene subnetworks that are enriched with specific SNPs and exhibit network rewiring across the 55 cell types. These subnetworks connect known and novel genes associated with a phenotype of interest offering novel hypotheses and prioritize validation experiments needed to improve our understanding of the role of regulatory variation in specific phenotypes.

## Results

### L-HiC-Reg accurately predicts contact counts in a large number of cell types

We previously developed HiC-Reg, an approach to computationally predict contact counts based on one-dimensional signals such as histone marks and transcription factor binding sites [18]. HiC-Reg performs well across chromosomes within the same cell type; however, predicting across cell types is still challenging and requires a large number of one-dimensional signals. We hypothesized that 3D genome conformation may be driven by different factors at different genomic loci. While a Random Forests prediction model should be able to capture multiple combinations of factors, a predictive model trained in a locus-specific manner can be more expressive and capture more nuanced dependencies than a global model for a whole chromosome. Therefore, we developed L-HiC-Reg, a “local” version of HiC-Reg that divides the chromosome into 1Mb segments and trains a separate model on each segment, as opposed to training on the whole chromosome (**Fig 1A** and **Methods**). Training on smaller segments also makes the prediction model much more scalable as it can be easily parallelized. L-HiC-Reg also uses a smaller number of discretized one-dimensional signals than originally used for HiC-Reg since these signals are widely available for many cell types enabling us to have a high coverage across diverse cell types. Briefly, to train L-HiC-Reg. we used features from seven experimentally measured and three computationally derived one-dimensional signals, together with genomic distance to represent a pair of regions. These datasets include the architectural protein CTCF, repressive marks (H3K27me3, H3K9me3), a mark associated with active gene bodies and transcriptional elongation (H3K36me3), active enhancer specific marks (H3K4me1, H3K27ac), a promoter mark (H3Kme3), cohesin component (RAD21), a general transcription factor (TBP) and DNase 1 (open chromatin). The histone marks were selected because they were the most frequently measured across the cell types in the Roadmap database, which we used to generate our compendium of predictions (**S1 Fig**). Datasets for CTCF, TBP and RAD21 were computationally derived from sequence and open chromatin (**Methods**) [30]. Similar to HiC-Reg, L-HiC-Reg predicts contact counts between 5kb pairs using the one-dimensional regulatory signals of histone marks, accessibility and architectural protein binding motifs. A pair of regions in L-HiC-Reg and HiC-Reg is represented by the features for both 5kb regions, as well as the average signals between the two regions (window signal) as initially proposed in TargetFinder [16]. The contact counts to be predicted were derived from high-resolution (5kb) Hi-C datasets from Rao et al (**Methods**) [31]. We applied trained L-HiC-Reg models on adjacent 1Mb regions using both original and imputed signals and predict in the same 1Mb region in a different cell type. We then concatenated the predictions for the entire chromosome.

**Fig 2:**
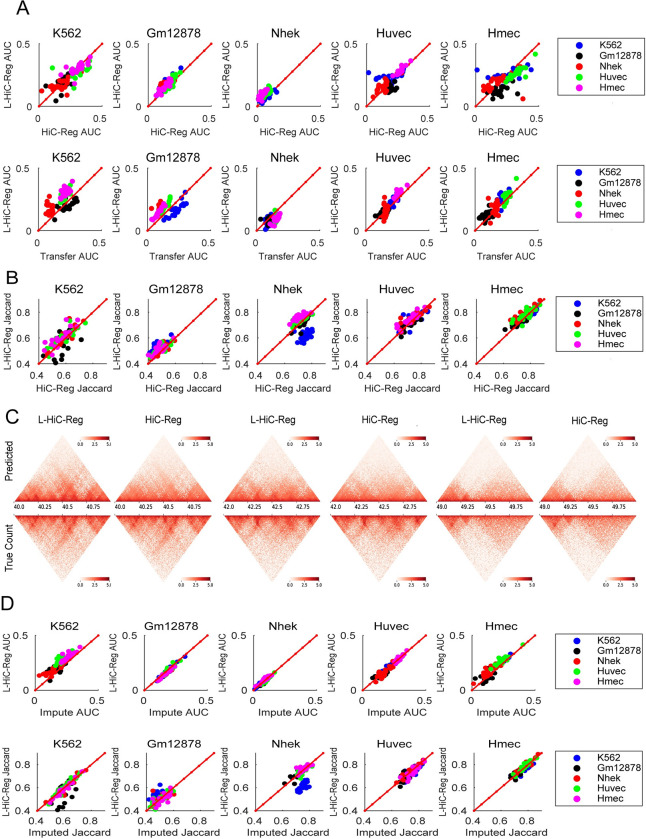
Evaluation of L-HiC-Reg predictions. **A.** Performance of L-HiC-Reg for count prediction between pairs of regions is assessed with the Area Under the correlation Curve (AUC) against HiC-Reg (top) and against transfer count (bottom). Each panel is a different test cell type and the color indicates the training cell type. **B.** Performance of L-HiC-Reg based on identified TADs from L-HiC-Reg predictions versus HiC-Reg predictions based on Jaccard coefficient similarity of TADs from true and predicted counts. Jaccard coefficient was used to assess the overlap of TADs found on the true counts with TADs found on the predicted counts by L-HiC-Reg (y-axis) and HiC-Reg (x-axis). **C.** Heatmaps of exemplar regions on chromosome 17 comparing Huvec L-HiC-Reg predictions and HiC-Reg predictions (top) against measured Huvec Hi-C data (bottom). **D.** Assessing predictions from measured versus imputed marks. AUC for L-HiC-Reg predictions generated from experimental one-dimensional datasets (y-axis) and imputed one-dimensional datasets (x-axis) in the test cell type (top). Jaccard coefficients comparing TAD recovery from measured (y-axis) and imputed data (x-axis) (bottom).

To test whether L-HiC-Reg recapitulates contact counts more accurately than HiC-Reg across cell types, we trained L-HiC-Reg and HiC-Reg models on the five 5kb-resolution Hi-C data sets from Rao et al each corresponding to a different cell line. We then generated all possible cross-cell type predictions for each chromosome resulting in 440 total predictions for each method (22 chromosomes x 20 cross-cell type combinations) [31]. We assessed the performance of L-HiC-Reg and HiC-Reg using distance-stratified Pearson’s correlation computed on the pairs from the test cell type, which we originally used for HiC-Reg [18]. We take the area under the distance-stratified correlation curve (AUC) as a measure of cross-cell type prediction accuracy. We found that in 270 of the 440 cross-cell type predictions, the AUC was higher for L-HiC-Reg than HiC-Reg (Kolmogorov-Smirnov (KS) p-value=0.0011). This suggests that in the majority of cross-cell type predictions, L-HiC-Reg recapitulated contact counts better than HiC-Reg (**Fig 2A**, **top row**). Specifically, L-HiC-Reg is significantly better than HiC-Reg with the test cell lines Gm12878 and Nhek (KS p-value < 0.05). Of these 270 instances, Hmec was most frequently used as the training cell line and Nhek as the test cell line (**S2A and S2B Fig**). Furthermore, chromosomes 2, 7, 16 and 15 benefitted most from L-HiC-Reg (**S2C Fig**). Full distance stratified correlation curves for cross cell line predictions from chromosome 10 further compares the performance of the two methods as a function of distance (**S3 Fig)**. Additionally, we compared the performance of L-HiC-Reg to a baseline model in which the count from the training cell type is used as the predicted count for the test cell type (“Transfer count”). L-HiC-Reg performed better than transfer count for 275 of the 440 cross-cell type predictions (KS p-value=1.5e-04). L-HiC-Reg also had higher performance than “Transfer count” in more cell lines than HiC-Reg (262 of the 440) suggesting that L-HiC-Reg is predicting more cell-type specific contact counts (**Fig 2A, bottom row**).

We next used the predicted counts from HiC-Reg and L-HiC-Reg to define topologically associating domains (TADs) by applying the Directionality Index (DI) TAD finding method [32]. We compared these TADs to TADs identified by DI on the true counts based on a metric derived from the Jaccard coefficient (**Methods**). The Jaccard coefficient is a number between 0 and 1, with 0 representing no agreement and 1 representing perfect agreement. We computed the Jaccard coefficients for all cross-cell type predictions on all chromosomes for L-HiC-Reg and HiC-Reg (**Fig 2B**). We found that the Jaccard coefficients were higher in L-HiC-Reg than HiC-Reg in 302 of 440 cross-cell type predictions, was significantly better in two of the five test cell types (Gm12878, K562, KS-test p-value <0.05) and was comparable in the other cell types. This suggests that L-HiC-Reg recapitulates TADs better than HiC-Reg when predicting in a new cell type. Visual inspection of the predicted matrices from the two methods for a 1Mb region in the HUVEC cell type (**Fig 2C**) also demonstrated a clearer TAD structure when using L-HiC-Reg than HiC-Reg. Taken together these results show that L-HiC-Reg can accurately predict contact counts and high-level features of chromatin architecture in new cell types.

### Prediction of Hi-C contact counts across multiple cell types with publicly available and imputed epigenomic datasets

To generate a compendium of high-resolution cell-type-specific Hi-C maps, we applied L-HiC-Reg Gm12878 models on publicly available epigenomic datasets for different cell types. Here, we considered cell types and tissues in the Roadmap Epigenome database for which a number of one-dimensional signals were available. These include DNase I for accessibility and six histone marks that are commonly measured (H3K27me3, H3K9me3, H3K36me3, H3K27ac, H3K4me1, H3K4me3), although often not within the same cell type. In particular, only 22 of the total 183 cell types had all six epigenomic signals and DNase I (**S1 Fig**). As the epigenomic dataset availability varies across cell types, it is difficult to make predictions from a model trained on features generated from a certain set of epigenomic marks that are not available in the test cell type. To overcome this limitation, we used imputed datasets in the test cell type to generate predictions in cases where the measured feature dataset is missing. Specifically, to acquire these imputations, we trained a version of Avocado [33] to predict read counts at 5kb resolution, as opposed to the original signal p-values at 25bp resolution, to match the resolution of our L-HiC-Reg models. Our Avocado model was trained using 559 experiments spanning 104 cell types and 33 assays, and we subsequently used the model to impute experimental readouts for histone modifications in the 55 Roadmap cell types and tissues for which DNase I accessibility was available. We chose not to use imputed DNase I because we needed the signal for motif feature generation of CTCF, RAD21 and TBP and for generating TF-gene networks in our subsequent analysis and interpretation pipeline (**Methods**).

To determine if we could accurately predict contact counts in a test cell type where all histone features are imputed (worst case scenario), we generated cross-cell type predictions for the 5 Rao et al. cell types using the imputed features in the test cell type as input to L-HiC-Reg models trained on the original measured values in the training cell type [31]. Based on the area under the distance-stratified correlation, the resulting performance was not significantly different from those generated from entirely experimental features (**Fig 2D**; KS test p-value=0.11). Additionally, we used the predictions based on imputed features to call TADs and found that the Jaccard coefficients from these TADs were also similar to the ones generated from experimental features (**Fig 2D,** KS test p-value=0.84). These results suggest that there is no significant deterioration in the quality of the predictions if imputed features are used. Finally, we applied our trained Gm12878 L-HiC-Reg models on measured and imputed chromatin mark signals, DNase I, and accessibility derived binding of CTCF, TBP, and RAD21 to all 55 cell types from the Roadmap project. We then performed a series of validation experiments for these predictions and leveraged the compendium to link SNPs to genes and downstream pathways as discussed in the following sections.

### L-HiC-Reg long-range interactions are validated by complementary experimental sources and associated with highly expressed genes

**Fig 3:**
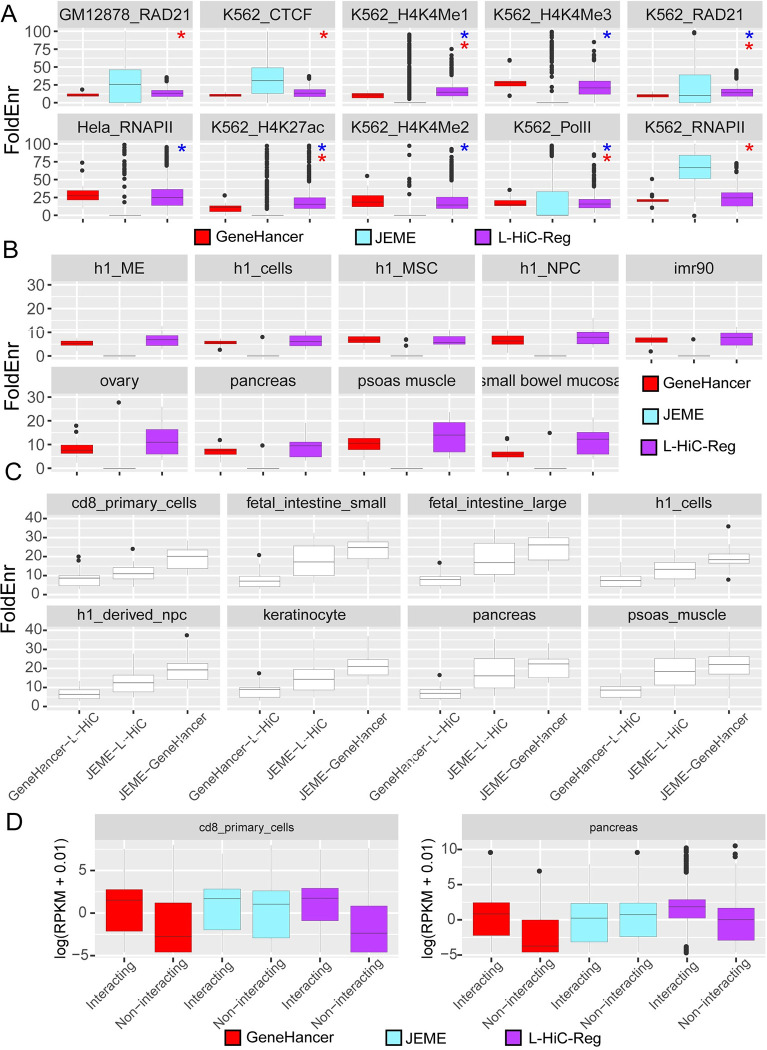
Performance of long-range interaction prediction approaches against experimentally derived datasets. **A.** Fold enrichment of JEME (cyan), GeneHancer (red) and L-HiC-Reg (purple) against gold standard ChIA-PET datasets. Blue asterisks indicate where L-HiC-Reg performed better than GeneHancer. Red asterisks indicate where L-HiC-Reg performed better than JEME. Predictions for each cell type from L-HiC-Reg and JEME were compared against each ChIA-PET dataset shown as a separate panel. GeneHancer is not cell type specific and had single set of predictions that were compared against all ChIA-PET datasets. Enrichment was calculated separately for each chromosome. The box plot shows the distribution of the enrichment values for each chromosome for a pair of predicted and ChIA-PET interactions **B.** Fold enrichment against capture Hi-C data. The predictions from L-HiC-Reg and JEME were compared to interactions from the capture Hi-C dataset matched by cell type. Enrichment was calculated separately for each chromosome. Colors correspond to different methods as in **A**. **C.** Comparing the fold enrichment of pairs of computational approaches GeneHancer and L-HiC-Reg, JEME and L-HiC-Reg and JEME and GeneHancer in matched cell types. **D.** Expression of genes (RPKM) with interactions compared to genes with no interaction in GeneHancer (red), JEME (cyan) and L-HiC-Reg (purple). L-HiC-Reg and JEME were matched for the cell type.

We defined long-range interactions as significantly interacting regions (loops) using a distance-stratified Binomial test [34] on the L-HiC-Reg predicted contact count matrices for each of the 55 cell types (**Methods**). Across 55 cell types, we identified between 681,889 and 1,481,911 significant (q-value <0.05) interactions with an average of 983,250 interactions. To evaluate these significant interactions, we compared them against interactions identified from complementary experimental assays: ChIA-PET and Capture-Hi-C (**Methods**). We obtained 10 published ChIA-PET datasets for different factors (RNA Pol II, CTCF and RAD21) and histone marks in multiple cell types [35,36]. We estimated fold enrichment of ChIA-PET interactions in the L-HiC-Reg-based interactions in each of the 55 cell types (**Fig 3A**). We did the same for two other computationally predicted resources, JEME [14] and GeneHancer [27]. GeneHancer is a network of enhancer-promoter interactions that scores potential enhancers and their interactions based on evidence from various publicly available datasets, such as expression Quantitative Trait Loci (eQTL) studies, functional annotation and chromatin accessibility. JEME uses a classification approach that incorporates information from multiple cell types to predict an interaction between a promoter and candidate enhancers. ChIA-PET interactions from all the 10 datasets were enriched (fold enrichment > 1) in the L-HiC-Reg, JEME and GeneHancer predictions (**Fig 3A**). High fold enrichment also correlates with significant P-values computed from a HyperGeometric test (**S4 Fig**). Additionally, L-HiC-Reg had a higher fold enrichment compared to JEME and GeneHancer in 7 out of 10 datasets (blue and red asterisk, **Fig 3A**).

We next compared L-HiC-Reg, JEME and GeneHancer predictions to several Capture Hi-C datasets from Jung et al., 2019, which profiled 27 cell types, 9 of which overlapped with the cell types we examined [37]. We compared directly with the matching cell type for all chromosomes. For GeneHancer, which does not predict cell type specific interactions, we considered the same set of interactions for each cell type. L-HiC-Reg interactions exhibit the highest fold enrichment for these datasets compared to JEME and GeneHancer, with the exception of h1 derived mesenchymal stem cells (h1_MSC) for which GeneHancer is better (**Fig 3B**). We also compared the predicted interactions from each of these three methods, L-HiC-Reg, Genehancer and JEME, to each other. All computational methods were mutually enriched among each other, with L-HiC-Reg exhibiting the highest enrichment in JEME interactions (**Fig 3C**). This is likely because L-HiC-Reg and JEME are both cell-line specific and random forest-based. The fact that there is a high enrichment among the different computational approaches is encouraging, especially since GeneHancer has a different underlying model.

Chromosomal looping is often associated with increased gene expression by bringing enhancers bound by transcription factors in close proximity to the promoters of their target genes. It has previously been experimentally demonstrated that transgenes inserted at interactions demonstrate higher expression values over all genomic distances, indicating that the presence of looping tends to increase transcriptional activity [38,39]. To test this property in our predictions, we linked significant interactions to genes in one or both of the interacting regions. Across the four cell types that were common to JEME and L-HiC-Reg and had RNA-seq data available, L-HiC-Reg linked an average of 10,341 genes over all the cell types, and such genes had a significantly higher expression (t-test P-value < 0.05) than genes that are not associated with significant interactions (**Figs 3D and S5**). GeneHancer linked 14,467 genes and also had a large difference between the genes with interactions versus genes that did not have interactions (t-test P-value<0.01). JEME linked an average of 5,810 genes across the cell types and the difference in expression between genes with and without interactions was not significant, likely because of the small number of genes linked by JEME. Taken together these results show that our compendium of L-HiC-Reg-based cell type-specific interactions is well supported by existing complementary assays and are of as good or higher quality than other computational predictions. While both JEME and L-HiC-Reg are cell type-specific, L-HiC-Reg produces a greater number of long-range connections to genes.

### Leveraging L-HiC-Reg long-range interactions to link regulatory SNPs to genes across diverse cell types

**Fig 4:**
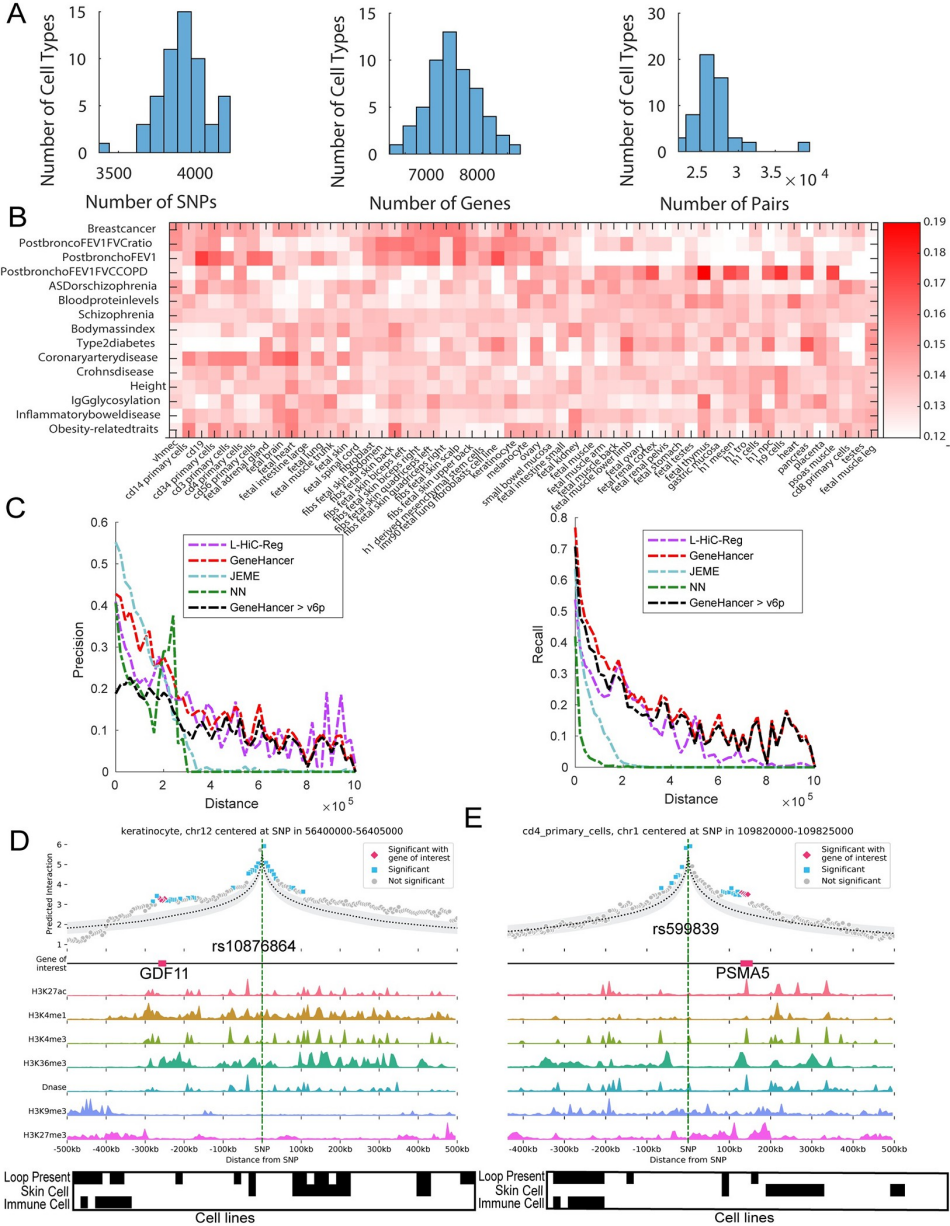
**A.** Distribution plots for all GWAS non-coding SNPs and SNPs in LD that were linked to genes across the 55 cell types (left), genes linked to SNPs (middle) and SNP-Gene pairs (right). **B.** Number of genes associated with SNPs (row and then column normalized to length one) across phenotypes (rows) and cell types (columns). Each entry is normalized such the rows are of length 1. Rows and columns are reordered using non-negative matrix factorization. **C.** Precision (left) and recall (right) of L-HiC-Reg, JEME, GeneHancer and nearest neighbor (NN) for eQTL SNP-gene associations as a function of distance (x-axis). **D.** Contact count and one-dimensional signals centered around the vitiligo-associated SNP rs10876864. The *GDF11* gene is highlighted on the Gene of interest track. The first row of the black-white heatmap below represents the presence(black)/absence(white) of the *GDF11*-rs10876864 interaction. The columns are cell types and tissues. The second row indicates which cell types are skin-related and the bottom row represents which cell types are immune-related. **E.** Contact count and one-dimensional signals for coronary artery disease-associated SNP rs599839. The *PSMA5* gene is highlighted on the Gene of interest track. The first row of the black-white heatmap below indicates the presence or absence of the *PSMA5*- rs599839 loop, the second row indicates which are the skin cell types and the third row indicates the immune cell types.

We next used our compendium of significant L-HiC-Reg interactions to link non-coding SNPs to genes in a cell-type-specific manner across the 55 cell types. We downloaded the NHGRI-EBI GWAS catalog, containing curated GWAS SNPs for thousands of phenotypes [1]. For a given GWAS, we defined a non-coding SNP as one that was labeled as intergenic and identified all SNPs in Linkage Disequilibrium (LD) with it (*R*^2^ = 0.8), resulting in a total of 23,116 GWAS non-coding SNPs and 364,893 SNPs in LD (**Methods**). We mapped all non-coding SNPs and SNPs in LD to genes using L-HiC-Reg significant interactions across all 55 cell types (**Methods**). Of the total 23,116 GWAS SNPs, we were able to map 35.16% to genes across our 55 cell types with an average 7,450 genes and around 30k interactions across cell types **(Fig 4A**). Of the 364,893 LD SNPs, L-HiC-Reg mapped 27.9% to an average of 9,549 genes in any cell type with ~40k interactions. In comparison, both JEME and GeneHancer mapped fewer proportions of SNPs (24.47% with JEME and 28.4% using GeneHancer). Similarly, for the SNPs in LD, L-HiC-Reg mapped a higher proportion of SNPs to genes (27.19%, **S6 Fig**) compared to JEME (18.7%) or GeneHancer (21.17%).

As SNP-gene associations could indicate regulatory relationships between the SNP and the gene by impacting its expression, we leveraged expression QTL (eQTL) datasets from the GTEx database [40] to assess the SNP-gene associations from each of the methods (**Methods**). We considered 4149 GWAS and 73662 LD SNPs for 15 phenotypes in the GWAS catalog with the largest number of SNPs (**Table 1**). The number of genes linked by L-HiC-Reg varied between 100-2400 across the phenotypes and was relatively stable across cell types (**Fig 4B**). For all GWAS SNPs in the NHGRI catalog associated with a gene, we calculated the proportion of predicted interactions that are also supported by eQTL SNP-gene associations (precision) and the proportion of eQTL SNP-gene associations in each predicted set (recall). As a baseline, to assess the importance of leveraging long-range interactions to link SNPs to genes, we also found the closest gene (nearest neighbor, NN) based on genomic distance to each GWAS and LD SNP and compared these SNP-gene associations to eQTL associations. To account for the fact that eQTL SNP-gene pairs are more likely to be close together and many of the predictions are between close regions, we stratified these two quantities by distance **(Fig 4C**). SNP-associations from L-HiC-Reg and GeneHancer had a higher precision than JEME for all distance bins compared, and GeneHancer had a higher precision than L-HiC-Reg for more distance bins (35 vs 16). Based on recall, GeneHancer was generally better than L-HiC-Reg with the exception of associations at 400-600k. Both methods were better than JEME for recall. The higher performance of GeneHancer is not surprising as it uses GTEx data, along with many other resources, to assign a score to an interaction. GeneHancer used GTEx data from versions lower than 6p (<6p) to generate its results, while we used v8 for this analysis. We therefore tested GeneHancer on GTEx data with version greater than 6p and found that its precision and recall decreased (**Fig 4C)**. L-HiC-Reg performed much better than GeneHancer on version greater than 6p (>6p) GTEx data, for precision but still slightly worse in terms of recall. JEME performs well in close-range interactions, but rapidly declines in precision and recall for longer-range interactions. Notably, all computational approaches perform better than the NN approach and, for distances greater than 200kb, very few NN genes are in eQTL (**Fig 4C**). Similar observations can be made for the set of LD SNPs (**S7 Fig**). Taken together, these results highlight the importance of leveraging long-range interactions to link SNPs to genes, particularly those predicted at >200kb distances.

**Table 1:** Statistics for 15 phenotypes analyzed using our framework. Shown are the total number of GWAS non-coding SNPs, total number of SNPs in LD with these SNPs, total number of SNPs (GWAS and LD) linked to genes, total number of genes linked to SNPs and total number of SNP-gene pairs.

Phenotype	GWAS	LD	SNPs Mapped	Genes mapped	Pairs
Autism spectrum disorder or schizophrenia	134	300	365	808	15439
Blood protein levels	154	2837	1975	1192	37676
Body mass index	467	10791	2533	1294	47762
Breast cancer	354	1697	644	1641	8074
Coronary artery disease	235	174	193	1036	1737
Crohn’s disease	170	6171	3181	1232	54566
Height	266	9530	3233	1451	37839
IgG glycosylation	223	4093	1266	760	28467
Inflammatory bowel disease	177	5113	2458	1349	34933
Obesity-related traits	319	2676	942	988	7094
Postbronchodilator FEV1	301	3721	556	568	6868
Postbronchodilator FEV1/FVC ratio	579	10003	563	979	4219
Postbronchodilator FEV1/FVC ratio in COPD	137	3883	235	167	1216
Schizophrenia	397	9714	3331	2378	72364
Type 2 diabetes	236	2959	1040	813	11508

Next, we manually verified several of the SNP-gene associations that L-HiC-Reg predicted using Capture Hi-C assays [41,42]. We obtained two large-scale Capture-Hi-C datasets that investigated GWAS SNP-gene interactions. One of these focused on blood cell traits and generated a Capture-Hi-C dataset in lymphoblastoid cells [41]. The second study used Capture-Hi-C in macrophages and studied immune and cardiovascular risk variants [42]. In addition to Capture-Hi-C datasets, these studies used a number of computational filters, such as motif disruption and chromatin state signal to identify SNP-gene associations. We filtered these SNP-gene associations based on whether interactions were distal which is defined as any SNP-containing region directly interacting with a probed promoter [41]. In our predictions, we found the SNP rs10876864 to loop to *GDF11* which was also identified by Cavalli et al., based on Capture-Hi-C, motif disruption and chromatin signals (**Fig 4D**). This SNP is associated with vitiligo, a skin disease, and *GDF11* is known to be involved in the regulation of skin biology. The black-white heatmap in **Fig 4D** shows which cell types the loop is present in (top row), which cell types are skin-related (middle row) and which cell types are immune (bottom). The L-HiC-Reg predicted count profile shows a significantly high value for this SNP and *GDF11*, compared to the background count distribution in one exemplar cell type (keratinocyte, gray shading, **Fig 4D**). Furthermore, inspection of the one-dimensional signals in the keratinocyte cell type showed a clear association of H3K4me1, an active enhancer mark, between the SNP and the gene. Similarly, we found an interaction between the SNP rs599839, associated with cardiovascular diseases, and the gene *PSMA5*, that was present among immune cell types (black-white heatmap, **Fig 4E**). Inspection of the predicted count profile and one-dimensional signals around this SNP in CD4 primary cells again highlighted the high interaction count for this interaction supported by one-dimensional signals such as DNase I and H3k4me3. Overall, these results offer experimental support for our predictions many of which are likely valid regulatory interactions based on the overlap with eQTL studies.

### Identifying downstream pathways impacted by regulatory variants

**Fig 5:**
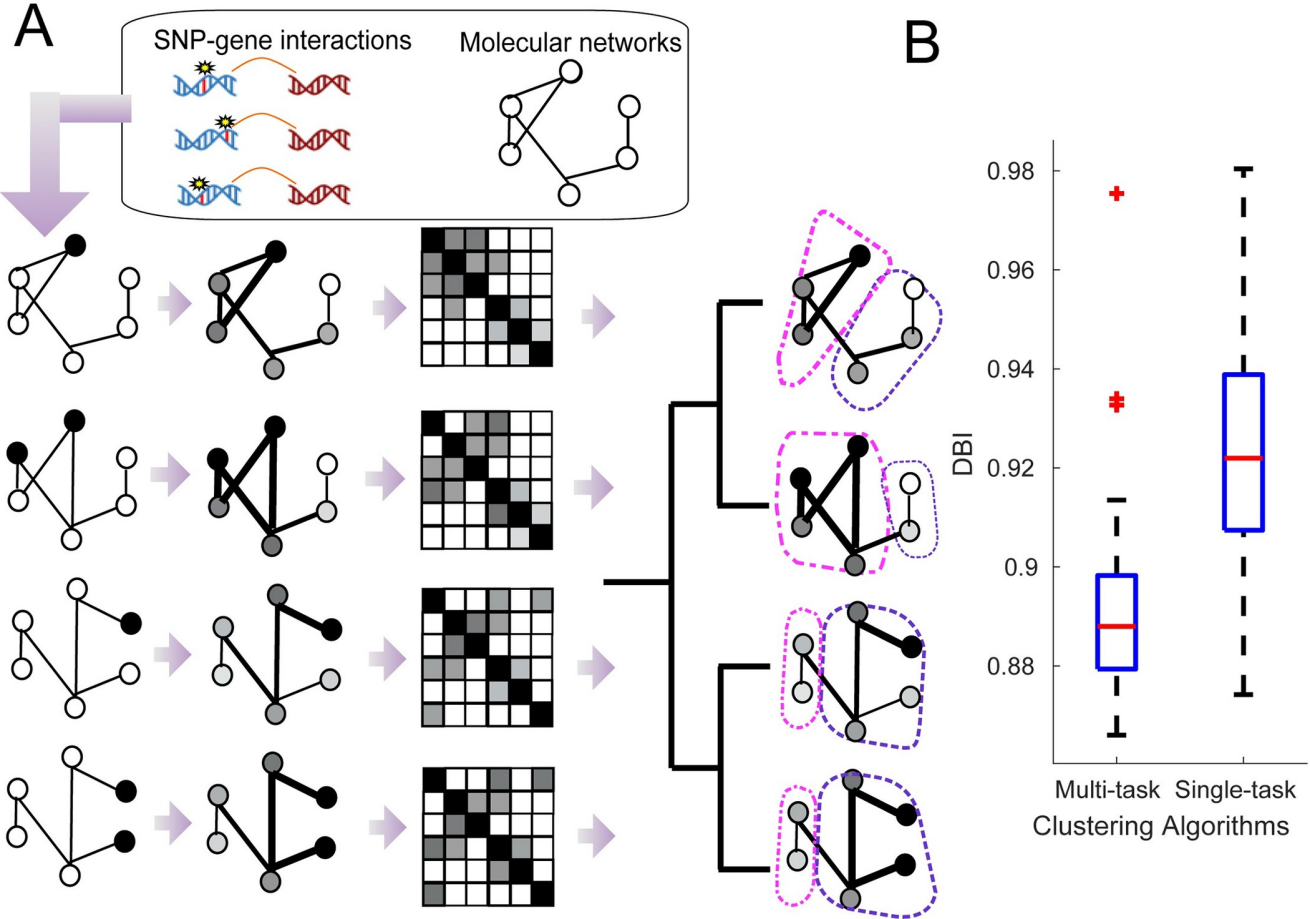
**A.** Overview of our Multi-task Graph Clustering (MTGC) method. Genes are scored based on their significant interactions with SNP-containing regions and mapped to a physical molecular interaction network. Two stage graph diffusion is performed to obtain a fully connected adjacency matrix with edge weights corresponding to the effect of SNP from one gene to another. The input into the multi-task graph clustering approach is a matrix for each cell type, the number of clusters and relationship tree for the cell types. The outputs are the matched gene clusters (dashed purple and magenta groups) corresponding to gene networks for each cell type. **B.** Comparison of the quality of clusters with Davies-Bouldin index for single task spectral clustering and our multi-task clustering approach (lower values are better) in the breast cancer phenotype for all cell lines and tissues.

Our results so far showed that we can successfully link regulatory variants to genes across diverse cell types. However, phenotypic variation is typically driven by groups of genes that interact in a larger unknown pathway and therefore interpretation of regulatory variation requires us to identify the impact of sequence variants at the pathway level. Systematic identification of pathways affected by a set of SNPs in a specific cell type is challenging because not all pathways are known and the extent to which they are preserved across different cell and tissue types is unknown. To examine the impact of a set of variants at the pathway level, we developed a graph-theoretic framework based on graph diffusion and multi-task graph clustering to simultaneously define gene subnetworks representative of gene pathways across different cell types (**Fig 5**). We first created a collection of cell-type-specific molecular interaction networks that combined protein-protein interaction networks, promoter proximal and distal transcription factor (TF)-gene interactions across each of the 55 cell types (**Methods**). The protein-protein interactions were context-unspecific while the TF-gene interactions were cell-type-specific because they were based on DNase I accessible motif instances and our long-range interactions. For a given phenotype, we scored a gene predicted to interact with a SNP based on the average -log(Pvalue) of its L-HiC-Reg interactions with SNPs (**Methods**). Such genes were called “direct hits.” We used graph diffusion to define additional downstream genes with no direct SNP interactions but with the greatest diffusion signal (top 1%) and created cell-line specific weighted graphs where the edge weight corresponded to the strength of a diffusion signal from the direct hits. We then applied a multi-task graph clustering approach, MUSCARI, to the graphs to identify different gene networks based on their connectivity to the direct hit genes (**Fig 5A**) [43]. Our multi-task graph clustering approach takes as input a relationship tree between the cell types and leverages these relationships while defining gene subnetworks for each cell type. This approach was found to be more advantageous for inferring gene clusters compared to single task clustering, as well (**Fig 5B**). We used the similarity of genome-wide H3K4me3 promoter signal between pairs of cell types and performed hierarchical clustering to obtain this tree (**S8A Fig**). The H3K4me3 signal-based tree was similar to a tree based on shared long-range interactions between pairs of cell types (**S8B Fig)** with a significantly high Fowlkes-Mallows index used to measure similarity between two hierarchical clusterings (**S8C and S8D Fig**). We applied our pipeline to each of the 15 phenotypes in the GWAS catalog with the most non-coding SNPs (**Fig 4** and **Table 1**). The phenotypes include a range of neuronal (autism spectrum disorder and schizophrenia), cardio-vascular (coronary artery disease), and auto-immune disorders (Crohn’s disease) as well as other diseases (breast cancer, Type 2 diabetes). Below we discuss two of these phenotypes, coronary artery disease and breast cancer. The results from our other phenotypes are available at https://regvar-networks.wid.wisc.edu/.

### Multi-task graph clustering for identifying cell type-specific subnetwork targets of non-coding SNPs in CAD

**Fig 6:**
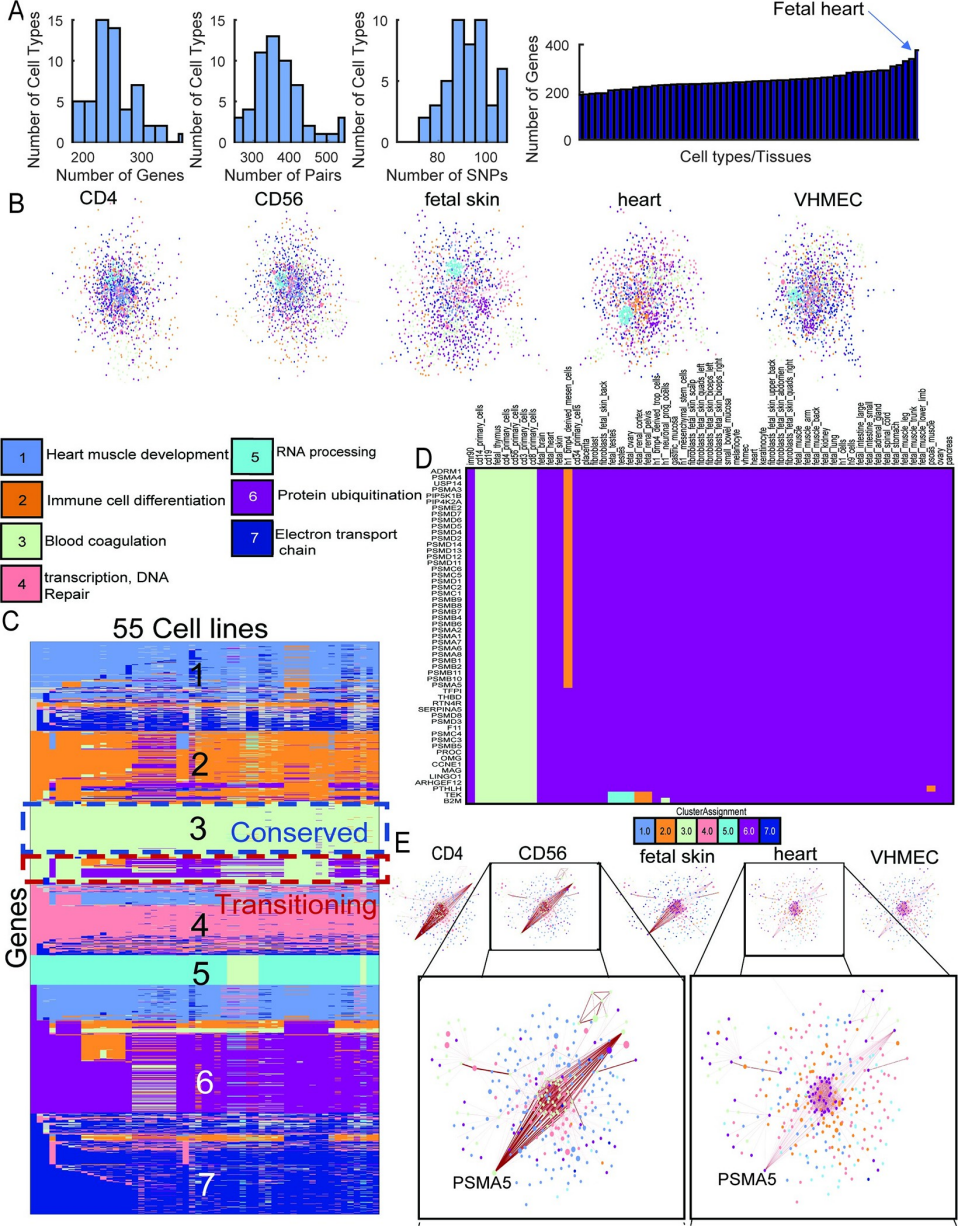
Interpreting regulatory variants in Coronary Artery Disease (CAD). **A.** Distribution of the number of genes linked to SNPs, number of pairs and number of SNPs linked to genes across tissues (left). Number of genes linked to SNPs for each cell type (right). **B.** Example networks with nodes (genes) colored by their clustering assignment and the major GO enrichment process for each cluster. **C.** Cluster assignments across the genes (rows) and cell types (columns). Rows are ordered according to cluster assignment in small bowel mucosa (first column), which is indicated in large font on the row groups. Some genes do not maintain their cluster assignment and are referred to as transitioning or differential. Conserved gene sets are those that have the same cluster assignment across all cell types. **D.** An example transitioning gene set. Rows are the genes and columns are the cell types with the color indicating cluster assignment (top). **E.** Networks of the genes in the set in **D** for representative cell types with (CD4, CD56) or without the interactions (heart) around PSMA5 with edge weight represented by edge thickness (bottom). The size of the node represents the node score after graph diffusion.

Coronary artery disease (CAD) is a type of cardio-vascular disease associated with thickening of arterial walls and is a major cause of disease in developed countries [44]. Recent GWAS of CAD have shown a significant portion of associated SNPs to lie in non-coding regions of the DNA [45,46]. Using L-HiC-Reg we mapped a total of 193 SNPs (141 GWAS and 52 in LD) of total 407 SNPs to 1036 genes across the 55 cell types with a total of 1737 interactions (**Fig 6A and Table 1**). Among the cell types that had the largest number of genes were fetal heart, skin and immune cell types like CD4 and CD34 (**Fig 6A**). After graph diffusion and selecting the top 1% of genes with the highest diffused score in each cell type, we had a total of 1866 genes across the cell types (1449-1821 genes in any cell type, **S1 Data**). We determined the optimal number of clusters (k) for MUSCARI based on modularity of clusters, each cluster corresponding to a gene subnetwork, defined across the 55 cell types and found k=7 to be best. We tested the clusters for enrichment of Gene Ontology (GO) terms in each of the clusters for the 55 cell types and analyzed the enrichment patterns with non-negative matrix factorization (NMF) to identify sets of GO terms associated with groups of cell types/MUSCARI clusters. NMF provided a faithful low-dimensional representation of the enrichment patterns with an explained variance of 0.97. Using NMF factor scores we selected the top GO terms and cell types/MUSCARI cluster for each NMF cluster (**S9 Fig** and **Methods**). The clusters exhibited enrichment in diverse processes including immune cell differentiation, heart development, and protein ubiquitination. **(Figs 6B and S9**). In particular, NMF identified a bi-cluster of GO terms relevant to CAD such as blood coagulation, wound healing, platelet activation and regulation of fluid levels that are associated with fetal heart and other muscle tissues in MUSCARI cluster 2 (**S9 Fig**). Dysfunctional coagulation has been shown to be associated with coronary artery disease [47]. Low mean platelet volumes are associated with worse outcomes for CAD patients [48]. Additionally, fetal heart is a top cell type for cluster 2 (**S9 Fig**).

We next examined the gene cluster assignment across the 55 cell types to identify genes that change their cluster assignment across cell types. Such genes are differentially connected across cell types and represent cell-line specific network components. We clustered genes into gene sets based on their gene assignment across cell types (**Methods**). Of the 1866 genes in the CAD phenotype, 968 genes were in a total of 44 transitioning gene sets and therefore differentially connected. Twelve of the 44 gene sets were associated with SNPs in at least one cell type indicating they were jointly targeted by these SNPs. We examined each of these gene sets for the correspondence between presence-absence patterns SNP-gene interactions across each cell type and the change in cluster assignment. Several of the 44 transitioning gene sets showed concordant changes in cluster assignment and SNP-gene interaction, including #196, #200 and #243 (**S10 Fig**). In particular, genes in set #243 were in cluster 6 for most of the cell types but transitioned to cluster 3 in the immune cell types (**Fig 6D**). This gene set included *PSMA5* and *PSB4* that had a number of CAD SNPs associated with them, specifically in the immune cell lines that transitioned (**S10A Fig**). This gene set also exhibits differences in the diffused edge weights between immune cell types and non-immune cell types, particularly around the *PSMA5* gene (**Fig 6D**, **bottom**). The association of *PSMA5* with one of the SNPs was also identified by a Capture-HiC experiment [42] (**Fig 4E**). Most of the genes in the transitioning gene set are in the ubiquitin-proteasome family of genes, which plays an important role in cardiovascular disease as elevated levels of ubiquitin are found in advanced coronary artery plaques [49]. While *PSMA5* has been shown before to associate with CAD GWAS SNPs, *PSMB4* is a novel prediction from our study. *PSMB4* has been shown to inhibit cardiomyocyte apoptosis and is a potential therapeutic target [50]. Genes in transitioning gene set #196 were in cluster 2 for most cell types but transitioned to cluster 1 in fetal brain, fetal heart, fetal skin and mesendoderm cells **(S10B Fig).** This gene set contains *PIM1*, which is specifically targeted by SNPs in the transitioning cell types. *PIM1* has been implicated in vascular smooth muscle cell proliferation in atherosclerotic plaques and is strongly upregulated in coronary artery disease [51]. Gene set #200 transitions to cluster 3 in a range of skin-related cell types such as fibroblasts, keratinocytes and melanocytes, as well as heart (**S10C Fig**). CAD-associated SNP rs6006426 specifically targets LIF in many of these cell types. LIF has been shown to be protective to the myocardium under various stressors such as ischemia, indicating it could be a therapeutic target in various heart diseases [52]. Taken together, our analysis of CAD GWAS SNPs demonstrates the utility of our network-based interpretation framework to pinpoint specific gene subnetworks that could be targets of different regulatory SNPs. These subnetworks included known and novel genes for CAD and represent plausible candidates for future validation studies.

### Identifying cell type-specific subnetwork targets of non-coding SNPs in breast cancer

**Fig 7:**
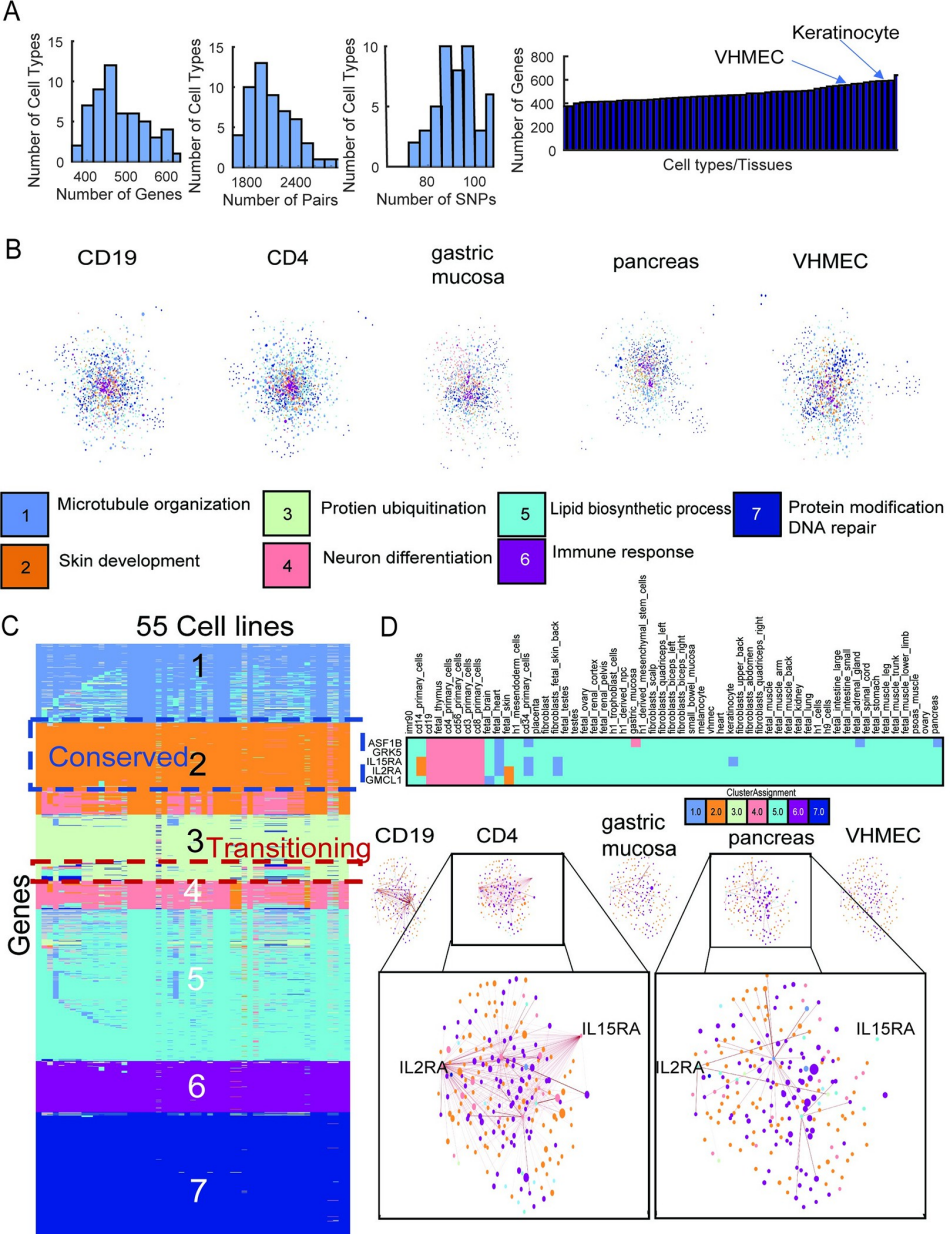
Interpreting regulatory variants in breast cancer. **A.** Distribution of the number of genes linked to SNPs, number of pairs (blue) and SNPs linked to genes across tissues (left). Number of genes linked to SNPs for every cell type (right). **B.** Example networks with nodes (genes) colored by their clustering assignment and the major GO enrichment process for each cluster. **C.** Cluster assignments across the genes (rows) and cell types (columns). Conserved and transitioning gene sets are indicated. **D.** An example transitioning gene set. Rows are the genes and columns are the cell types with the color indicating cluster assignment (top). Networks of the genes in this set (triangles) and their nearest neighbor target genes (circles) with edge weight represented by edge thickness (bottom) for five representative cell types where the interaction is present (CD19, CD4) or absent (pancreas, vHMEC).

As another application of our analysis pipeline, we considered non-coding SNPs identified in breast cancer GWAS studies. Using L-HiC-Reg we mapped a total of 644 SNPs (158 GWAS and 486 in LD) of total 2034 SNPs to 1641 genes across the 55 cell types with a total of 8074 interactions (**Fig 7**). Among the cell types that had the greatest number of genes targeted by SNPs were various epithelial cells such as keratinocyte and variant human mammary epithelial cells (vHMEC), which exhibit a pre-breast cancer-like phenotype (**Fig 7A**). After graph diffusion and selecting the top 1% of genes with the highest diffused score in each cell type, we had a total of 1864 genes across the cell types (1241-1797 genes in any cell type, **S2 Data**). NMF of the GO terms associated with the breast cancer clusters and cell lines (explained variance = 0.985), identified a diverse range of processes such as skin development, immune response and DNA repair for these clusters (**Figs 7B and S11**). MUSCARI cluster 6 was enriched for glucose metabolic processes and MUSCARI cluster 4 was enriched for response to glucose stimulus (**S11 Fig**). Glucose has been shown to promote breast cancer aggression and reduce the efficacy of chemotherapy drugs [53]. Examining the cluster assignments across cell lines we identified genes with conserved and transitioning cluster assignments across cell types (**Fig 7C**). Of the 17 total transitioning gene sets, 14 were associated with breast cancer SNPs and gene set #225 had several SNPs associated with genes in the immune cell types (**S12 Fig**). The genes in transitioning gene set #225 are in cluster 5 for most cell types, but transition to cluster 4 in the immune cell types (**Fig 7D**). These genes exhibit a higher degree of connectivity, particularly around the IL15RA and IL2RA genes, in the immune cell types compared to other cell types (**Fig 7D**). Furthermore, IL15RA and IL2RA are predicted to be targeted by several breast-cancer associated SNPs in cd19 and cd4 primary cells, as well as in vHMEC (**S12 Fig**). *IL15RA* encodes the interleukin 15 cytokine receptor, and knockdown experiments have confirmed that the protein plays a role in cell growth and apoptosis in breast cancer cell types [54]. *IL2RA* has also been associated with an increase in breast cancer risk, specifically postmenopausal, as well as mortality [55]. Taken together, these results suggest that long-range regulation with cytokine receptor genes in immune cell types may be important in breast cancer.

## Discussion

Interpreting non-coding variation and how it impacts phenotypic variation is a significant challenge because of our limited knowledge of which genes and pathways these variants act upon [56]. Long-range regulatory interactions between distal sequence elements and genes are emerging as a major mechanism by which variants can impact gene expression [4–7]. However, high-resolution maps of such interactions are missing for most cell types and biological contexts due to the cost of generating high-resolution Hi-C datasets. Furthermore, tools to identify pathways that are impacted by a set of variants in a cell type specific manner are limited.

Here, we presented a computational pipeline to first generate *in silico* Hi-C maps across a large compendia of cell types and then leverage these maps within a graph-theoretic framework to define gene subnetworks targeted by sets of sequence variants. Our pipeline comprises L-HiC-Reg, a tool to use experimental and imputed signals from the Roadmap epigenomics database to predict high-resolution Hi-C maps across multiple cell types, and multi-task graph clustering to simultaneously define gene subnetworks across multiple cell types. Compared to our previous tool, HiC-Reg, L-HiC-Reg is more scalable and generalizable to new cell lines. Using our approach, we were able to link a higher proportion of variants to genes compared to existing approaches, many of which are supported by auxiliary experimental sources such as eQTL, Capture-Hi-C and ChIA-PET datasets. We used our approach to predict cell-type-specific pathways across 55 cell types for 15 different phenotypes. With our graph-theoretic framework, we were able to find gene sets that change their connectivity across cell types and harbor sequence variants for a phenotype.

Our computational pipeline and associated resources greatly expands on previous efforts for leveraging long-range interactions to link sequence variants to genes. In particular, compared to some resources [27], our resource is context-specific comprising predicted loops and count matrices for individual cell types. Second, our regression-based model, L-HiC-Reg predicts contact counts for every genomic pair within a 1 Mb distance, as opposed to methods which directly generate the set of interactions [14]. By predicting counts, we offer the end-user the flexibility to apply different statistical tools to define interactions and also identify higher order units of organization such as topologically associating domains (TADs). Finally existing approaches for interpreting regulatory variation using long-range interactions (Cavalli et al., 2019) [41] typically operate at the level of individual SNP-gene pairs. Our pipeline leverages cell-type-specific promoter proximal and long-range regulatory interactions and cell type non-specific protein-protein interactions to predict gene subnetworks that are targeted by sets of variants in a cell-type-specific manner.

Functional validation of interactions between regulatory variants and genes is challenging using both experimental and computational methods because of the large number of possible associations. By overlaying variants on a molecular network, with nodes and edges reweighted by their propensity to participate in long-range interactions with SNPs, we were able to narrow possible gene candidates that could be targeted by a SNP. We leveraged cell-type-specificity of our networks to identify genes that change their clustering structure and therefore network connectivity across cell types and found many of these gene sets to have sequence variants. Furthermore, correlating the change in network connectivity with the presence-absence pattern of the sequence variant-gene interaction helped narrow down the list of candidate gene-variant interactions that could be experimentally validated.

We used our resource to examine non-coding variants in 15 different phenotypes spanning different disease and normal phenotypic traits. For each of the phenotypes we defined cell type specific gene targets and subnetworks across 55 different cell types. Our predictions are available for download at https://github.com/Roy-lab/Roadmap_RegulatoryVariation, with a user-friendly interface at: https://regvar-networks.wid.wisc.edu/. Comparison of the subnetworks across cell types showed that most genes do not change their network connectivity. However, changes in network connectivity were often associated with their tendency to link to SNPs. One such example was for coronary artery disease (CAD) that included a set of 6 genes, that were predicted to interact with CAD SNPs in immune cell types. We found a large number of connections with the genes *PSMA5* and *PSAP*, which were depleted in the other cell types. While *PSMA5* was shown previously to link to CAD SNPs by a previous Capture-Hi-C study [41], *PSAP* is novel to our study. Taken together, these results suggest that our approach can identify relevant gene subnetworks that are associated with SNPs for a phenotype of interest and can be used to investigate the effects of non-coding variation.

Our work can be extended in a number of ways. Here, we relied on regulatory interactions based on the presence of sequence-specific motifs, which did not include transcription factors (TFs) that do not have known motifs. One direction of work is to expand our TF-target connections by leveraging expression-based network inference methods [57]. Furthermore, much of our work relied on cell types and primary tissues with available accessibility and histone modifications. Single cell RNA-seq and accessibility datasets are increasingly growing and expanding our ability to define cell types in diverse tissue and organ systems [58]. Therefore, another direction of work is to extend our approach to single cell omic datasets to both define long-range interactions, gene regulatory interactions, and identify cell-type-specific gene subnetworks that are potential targets of regulatory variants. The resolution of our SNP-gene long-range associations are also limited to the resolution of the Hi-C data (5kb) that we used, and therefore we cannot distinguish multiple SNPs overlapping the same 5kbp bin. Although we incorporate sequence based on accessible motif instances, it is limited to known motifs of select proteins. As future work, we plan to combine sequence more extensively together with the continuous regulatory signals to more directly assess the impact of the SNP on the strength of interaction. Recent deep learning methods for predicting Hi-C count from DNA sequence would be a possible approach towards this direction [19,20,59]. Our work relied largely on GWAS to define our set of variants. Another direction of work would be to consider a broader range of sequence variants, for example from whole genome sequencing of clinical phenotypes [60,61]. As more genomes, transcriptomes and epigenomes of individuals and populations become available, approaches such as ours should be helpful to improve our ability to interpret non-coding variants and their impact on individual genes as well as on entire pathways.

## Materials and methods

### L-HiC-Reg

L-HiC-Reg is based on HiC-Reg, a Random Forests regression approach that predicts contact counts using one-dimensional regulatory genomic data sets, e.g. histone modifications and architectural proteins, and chromatin accessibility [18]. Because the focus of L-HiC-Reg is to generalize across a large number of cell types, we made several modifications to the original HiC-Reg approach which improved performance. First, we used models trained on a smaller number of datasets to maximize the number of cell types for which we can make predictions. Second, L-HiC-Reg uses discrete features (described below) compared to HiC-Reg which uses continuous features to make the features more comparable across cell types in training and prediction tasks. Lastly to train the L-HiC-Reg models, we first segmented a chromosome into non-overlapping 1Mb segments and trained a Random Forests regression model for each adjacent 1Mb segment using high-resolution (5kb) Hi-C SQRTVC normalized data downloaded from Rao et al [31], although based on our previous work, other normalization methods such as Knight-Ruiz matrix balancing or Iterative Correction and Eigen vector decomposition (ICE) can be used as well. For a given 1Mb segment, the training set included all 5kb region pairs in which one or both of the 5kb regions in the pair was inside the 1Mb segment (**Fig 1A**). We then made predictions on the same 1Mb region in a test cell type. We concatenated predictions from multiple 1Mb models for the entire chromosome. If the two 5kb regions of a pair were in different 1Mb segments, the average from the two models was used. We trained Random Forests regression models for each of the human autosomal chromosomes. We used all five cell lines from Rao et al., as training and test cell lines for our initial evaluations. For generating predictions in the Roadmap cell types, we used Gm12878 models as this had the highest depth data. Additionally, Gm12878 performs well in predictions across the five cell lines. When comparing predictions from different training cell lines to H1 cell lines with real high resolution data from naïve hESC cells, Gm12878 was the second best performer [62] (**S13 Fig**).

### Feature extraction and pair representation

#### Datasets

We used the following datasets to generate features for L-HiC-Reg: DNase1-seq, H3K27ac, H3K27me3, H3K36me3, H3K4me1, H3K4me3 and H3K9me3. We selected these signals because they were measured in most cell types and tissues in the Roadmap Epigenome Mapping consortium database. We obtained these datasets from the ENCODE project for the five cell types with high resolution Hi-C data in Rao et al.: K562, Gm12878, Hmec, Huvec and Nhek that we used for training and testing [31]. We used 55 cell types from the Roadmap database that had measured DNase I profiles as our test cell types. However, only 22 cell types had all the feature datasets measured; therefore, we imputed missing values described below (**Imputation of epigenomic signals**). In addition to the seven datasets above, we used measured DNase I-seq to derive accessible binding motifs for CTCF, RAD21 and TBP, using PIQ [15,30] since ChIP-seq data is not available for these proteins in the Roadmap database. We obtained the raw fastq files from ENCODE [63] and the Roadmap project (Roadmap Epigenomics Mapping Consortium et al., 2015)[26], and we aligned reads to the human hg19 assembly using bowtie2 [64]. Reads aligned to a locus were obtained using SAMtools [65] and input to BEDTools [66] to obtain a base pair level read count which was then aggregated in a 5kb region. We then normalized the aggregated signal by sequencing depth and collapsed replicates by the median. Finally, we discretized the feature values into 20 bins using a k-means algorithm for each signal and matched the discrete levels across cell lines. Alternate discretization features such as quantile binning resulted in over smoothing the data.

#### Imputation of epigenomic signals

We generated imputations using a modified version of Avocado [33]. Avocado is a deep tensor factorization method that organizes a compendium of epigenomic data sets into a three-dimensional tensor with the axes being cell types, assays, and genomic positions. The learned latent representations for each axis, and the neural network predictor, are trained jointly using a mean-squared-error objective function on the task of predicting observed values within the tensor. The trained model is then used to make predictions for all experiments, regardless of whether they have corresponding experimental data. Avocado’s original formulation made predictions at 25bp resolution and employed genomic latent representations at 25bp, 250bp, and 5kb resolution. Because the data in this work was at 5kb resolution, all latent representations and the output resolution of the imputations, were changed to 5kb resolution. Other than the change in resolution, the Avocado architecture and training scheme was the same procedure as outlined in Schreiber et al, 2020 [33].

#### Feature extraction

L-HiC-Reg makes predictions for each pair of 5kb regions. As described previously [18], we first generated features for each 5kb bin and represented each pair of regions using the features of the two regions and the window region between them. A ChIP-seq signal was represented as the average read count aggregated into a 5kb bin. To account for overall differences in signals across cell types, we discretized each 5kb ChIP-seq signal using k-means clustering with k=20 for each feature. For TBP, RAD21 and CTCF accessible motifs we used the number of motif instances with a PIQ purity score greater than 0.50 for each 5kb bin as features.

Each 5kb region was represented as a 10-dimensional feature vector, each dimension corresponding to one of the 10 genome-wide datasets (6 histone ChIP-seq, DNase I-seq and the 3 DNase I-seq filtered motif instance counts). The feature value for the window region for a ChIP-seq or DNase 1-seq feature is the mean value of the signal in the intervening bins, followed by discretization. For the motif count features, the window feature value is the number of motifs divided by the number of 5kb distance bins in the window. Each pair’s features included two 10 dimensional feature vectors for each region concatenated with the feature vectors of the window region between the two regions to obtain a feature vector of size 30 [16].

#### Generation of contact counts in 55 Roadmap cell types

We used the 5 L-HiC-Reg models trained on the Rao et al cell types to generate the count predictions in the 55 cell types for each 1MB region [31]. Our final analysis was done on the predictions from the model trained on the Gm12878 cell line, which is the dataset with the highest sequencing depth. For a region-pair that spans two 1MB bins, we will have two predictions; we take the average of these predictions.

### Binomial method to call significant interactions

In order to determine significant interactions in the 55 Roadmap cell types, we adopted the binomial method used in Duan et al., 2010 [34]. The probability of observing a given interacting pair of 5kb bins exactly *k* times is computed via the binomial distribution

P(K=k)=(nk)mk(1−m)n−k

where *m* is the probability of observing any interaction and is assumed to be uniform and *n* is the total number of observed interactions. Thus, the probability that each pair is observed at least *k* times is

$$Pvalue=\sum_{i=k}^{n}P(K=i)$$

To control for the distance effect, we compute the p-values for different distance bins in increasing intervals of 5kb up to 1Mb. We also adopt the same multiple testing correction procedure as Duan et al., 2010, using the Benjamini-Hochberg method to estimate false discovery rate (FDR), and define the q-value as the minimal FDR threshold at which a given score is deemed significant.

### Evaluation metrics

#### Distance stratified Pearson’s correlation

To assess the quality of count prediction of L-HiC-Reg, we used Pearson’s correlation of predicted contact counts and true contact counts stratified by genomic distance as described previously [18]. This was done by grouping pairs of regions based on their genomic distance and calculating the Pearson’s correlation of predicted and true contact counts in each 5kb distance bin up to 1 MB. To easily compare the performance between L-HiC-Reg, HiC-Reg, transfer count and predictions generated from experimental and imputed data, we summarized the distance-stratified Pearson’s correlation curve into the Area Under the Curve (AUC) as described in Zhang et al [18]. Higher AUC indicates better performance.

#### Identification of TADs and TAD similarity

To identify topologically associated domains (TADs), we applied the Directionality Index (DI) method described in [32] using a genomic window of 2Mb on both measured and predicted contact counts at a resolution of 5kb. We compare the similarity of TADs identified from the predicted counts (both HiC-Reg and L-HiC-Reg) and true counts for each chromosome and combination of train and test cell type as described previously in Zhang et al [18]. Briefly, we matched a TAD found in the true count data to a TAD found in the predicted counts based on the highest Jaccard coefficient. The Jaccard coefficients for each match were averaged across all TADs from the true counts. We repeated the process for each TAD from the predicted counts to a TAD in the true count and averaged the Jaccard coefficient across TADs from the predicted counts. The overall similarity between TADs was then the average of these two averages.

#### Comparison of L-HiC-Reg predictions to computational and experimental approaches

From our predictions in the 55 cell types, we obtained significant interactions using the Binomial method (discussed above). We compared these significant interactions to datasets from two types of experimental assays (1) cell type specific Capture Hi-C data [37] and (2) 10 ChIA-PET data sets [35,36]. The Capture Hi-C experiments were performed on 27 cell types, of which 9 overlapped with the cell types for which we generated predictions. The ChIA-PET datasets included PolII in HeLa, K562 and Gm12878 cell types, CTCF and TAD in the K562 and Gm12878 cell types and multiple chromatin marks in K562 and Gm12878 cell types [35]. The Capture Hi-C datasets were tested in a cell type specific manner while the ChIA-PET were considered non-cell type specific and tested all of the ChIA-PET datasets against all of the 55 cell types.

Our metric for evaluating these genome-wide maps is fold enrichment, which compares the fraction of significant interactions identified from L-HiC-Reg that overlapped with experimentally detected measurements, against the fraction of interactions expected by random chance. We mapped experimental (Capture Hi-C or ChIA-PET) interactions onto the pairs of regions used in L-HiC-Reg by requiring one region of an interaction from the experimental dataset to overlap with one region, and the other experimental region to map to the second region. Fold enrichment is defined as $\frac{q/k}{m/S}$, where *q* is the number of significant interactions from L-HiC-Reg that overlap an interaction in the experimental data set, *k* is the total number of L-HiC-Reg significant interactions, *m* is the total number of interactions in the experimental dataset that can be mapped to any of the 5kb pairs used for L-HiC-Reg, and *S* is the total number of possible 5kb pairs in the universe. A fold enrichment greater than 1 is considered enriched.

We also compared our results to two computational approaches (1) GeneHancer [27], a non-cell-type-specific resource that scores enhancer-promoter interactions based on various public datasets such as chromatin accessibility, eQTL and functional annotations and (2) JEME [14], an approach that integrates chromatin accessibility, histone modifications and expression across a large number of biological samples to predict enhancer-promoter interactions using a binary classification framework. JEME first constructs a LASSO regression model around each promoter to identify candidate enhancers using expression and histone marks and then uses sample-specific errors and features to train a Random Forests model to predict enhancer-promoter interactions. We compared these methods to L-HiC-Reg using the fold enrichment metric described above.

#### Mapping significant interactions to genes and expression analysis

We mapped either end of the significant interactions in our compendium to genes using the Gencode v10 (2012) TSS annotation. A 5kb region in a pair can be associated with a gene if it overlapped within +/-2500bp of the TSS. Any gene that was associated to at least one significant interaction was considered “interacting” while any gene not mapped to any significant interaction was considered “non-interacting.” We downloaded RNA-Seq data from the Roadmap database which contained the RPKM values for 57 epigenomes. Of the 55 cell types, 14 cell types also had expression. We compared the log(RPKM) between interacting and non-interacting genes in these 14 cell types using a T-test. We also performed this analysis for GeneHancer and JEME interactions.

#### Mapping regulatory variants to genes with long-range interactions

We downloaded GWAS SNPs from the NHGRI-EBI GWAS catalog v1.0.2. We determined which GWAS SNPs were in non-coding regions based on whether they were annotated as “intergenic” or not. For all of these GWAS SNPs, we determined the SNPs that were in LD with the GWAS SNP with the tool LDproxy using an R^2 cutoff of 0.8 [67]. We identified significant interactions where the 5kb bin contains a coding gene promoter and the other bin contains a non-coding SNP in any of the 55 Roadmap cell types.

#### Evaluating predicted interactions based on eQTLs

We used curl to query the GTEx v8 database using all non-coding GWAS variants in the NHGRI catalog [40]. This results in the set of all genes in eQTL with a variant, producing eQTL-based SNP-gene pairs. Predicted SNP-gene interactions for L-HiC-Reg, JEME and GeneHancer were determined using the approach described in “Node Scoring” section of methods. We evaluated the overlap between SNP-gene interactions from L-HiC-Reg, GeneHancer, and JEME with eQTL-based SNP-gene pairs using (1) precision and (2) recall for the GWAS and LD variants. We also compared to a baseline method, nearest neighbor (NN), which is the closest gene neighbor to a SNP by genomic distance. A predicted SNP-gene pair was considered overlapping with an eQTL-based SNP-gene pair if the SNP had a predicted interaction with the same gene. Precision is calculated as the total number of predicted interactions overlapping SNP-gene pairs in eQTL over the total number of predicted interactions. Recall is calculated as the total number of predicted interactions overlapping SNP-gene pairs in eQTL over the total number of SNP-gene pairs in eQTL. These ratios are averaged over the phenotypes and cell types.

### Examining the downstream effects of non-coding variants on cell-type-specific gene networks

We developed a graph-theoretic framework to define gene subnetworks targeted by sets of SNPs for a phenotype of interest. We first assembled 55 cell type specific gene networks (See **Network generation**). Nodes on each network were scored based on their long-range interactions to non-coding SNPs (See **Node scoring**). The network nodes and edges were reweighted using graph diffusion (**Node and Edge Diffusion**) to define the combined effect of variants on genes based on the network structure. Finally, we applied multi-task graph clustering to define groups of genes on the network based on their reweighted connectivity across all 55 cell types.

#### Network generation

We downloaded a protein-protein interaction network (PPI) from STRING version 9.1 [68]. To obtain high-confidence edges, we considered only edges with a STRING confidence score > 0.95. To obtain cell type specific proximal networks, we downloaded motifs for 537 transcription factors from JASPAR [69]. We applied PIQ to find accessible motifs using the Roadmap DNase I data in all 55 cell types [30]. We selected accessible motifs with a PIQ score greater than 0.9. We generated a TF-target regulatory network by mapping the accessible motifs to gene promoters defined using 2500bp around the transcription start site (TSS) using Gencode v10 TSS annotations. Finally, we incorporated cell type specific, distal TF-target pairs via our long-range regulatory interactions. For a given cell type, we took the significant interactions with one region overlapping a gene TSS as defined by the Gencode v10 TSS annotations. We searched for a motif in the other region of the interacting pair using the same criteria as the proximal network. For both the proximal and distal networks, we took the top 5% of edges defined by the motif instance purity score. Finally, we merged the PPI network, and the proximal and distal TF-gene network to generate a cell-line specific network for each of the 55 Roadmap cell types.

#### Node scoring

For a given phenotype, we scored genes in a particular cell type based on whether they were connected to a non-coding GWAS SNP or an LD SNP via long-range interactions for that cell type. For each significant interaction, we matched one of the 5kb regions to a gene using the TSS (as described in “**Mapping significant interactions to genes and expression analysis**”) and the other region to a SNP if its genomic coordinate was within the 5kb region. There could be multiple SNPs associated with a phenotype within each bin, however our downstream methods (see: “Node and Edge Diffusion”) only require that we know which 5kb bins have SNPs and are associated with gene(s) in the predicted long-range interactions. The score of a gene was defined as the average -log(q-value) of all significant SNP-gene interactions involving that gene in a given cell type. Such genes were called “direct hits”. If a gene did not participate in a long-range interactions with SNPs, its initial score was set to 0.

#### Node and Edge diffusion

The genes directly interacting with a SNP (direct hits) described above may not fully capture all of the downstream effects of non-coding variation of a given phenotype, which might be broader and facilitated by an underlying molecular network. Therefore, we applied a two-step graph diffusion approach. First, we applied node diffusion using the direct hit genes as input nodes. Following node diffusion, we reweighted the edges using edge diffusion to obtain cell-type specific weighted graphs. Our two-step diffusion approach to obtain weighted graphs was motivated by the relatively small number of nodes as direct hits which could result in very low edge weights for clustering. Node diffusion measures the influence of the input genes on all other genes in the network based on their global network connectivity. We used the regularized Laplacian kernel to estimate global network connectivity [70]. This kernel is defined as *K*_*L*_ = (1+*λ*_*L*_)^−1^ where, *L* is the symmetric normalized Laplacian and is defined as *L* = *I*−*D*^1/2^*AD*^1/2^, where *A* is the adjacency matrix of the gene interaction network *G*, and *D* is a diagonal matrix giving the degree of each node. *λ* is a hyper-parameter specifying the kernel width that determines how fast information on the source/input nodes diffuses to other parts of the network. The diffusion score for a gene *g*_*i*_ is computed by *V* = *K*_*L*_∙*Q*, where *Q* is a 1-dimensional vector of input node scores, *V* is a vector of diffusion scores, (*v*_1_, *v*_2_,⋯,*v*_*n*_) and *n* is the total number of nodes in the graph. We tested different values of *λ*, {0.1, 0.5, 1, 5, 10}, using leave-one-out cross-validation on all 55 cell types and several phenotypes (Autism spectrum disorder or schizophrenia, breast cancer, coronary artery disease and Crohn’s disease). Treating the direct gene hits as labeled data, we scored each value of *λ* using the area under the receiver operating character characteristic curve (AUROC) computed from leave one out cross-validation. For each fold, we left one of the direct hits at a time, carried out diffusion and used the diffusion score when it was not included in the input set to predict its label. At a diffusion score *v*_*i*_, precision is computed as the fraction of all genes with score ≥ *v*_*i*_, that are in the direct hit set, and recall is computed as the fraction of all the genes in the direct hit set that have score ≥ *v*_*i*_. In general, *λ*≤1 performs the best across cell types and tissues, with minimal difference in AUPR between *λ* = 0.1,0.5,1. *λ* = 10 rarely performs the best **(S14 Fig)**. Therefore, we decided to use *λ* = 1 across cell types and phenotypes.

For our second step, edge diffusion, we used an insulated heat diffusion kernel [71,72] to estimate the influence of a node on its neighboring nodes based on their global connectivity. The insulated heat kernel is defined as *K*_*H*_ = β(*I*−(1−*β*)*W*)^−1^. *W* is a transition matrix defined as *W* = *AD*^−1^, where *A* is the adjacency matrix of the gene interaction network, and *D* is a diagonal matrix of node degrees. The parameter *β* specifies the retention rate of the kernel and was set to a value of 0.3 in this study after testing several values (0.1, 0.3, 0.5, 0.9) during graph diffusion and calculating the Davies-Bouldin index (DBI) after clustering (**S15 Fig**). We define a diagonal matrix *D*_*V*_, where an element of the matrix *d*_(*i*,*i*)_ is the diffused nodes score *v*_*i*_ from the node diffusion step. We computed the final weighted adjacency matrix as *H* = *K*_*H*_∙*D*_*V*_. We then obtained a cell-type specific submatrix from the nodes that were scored pre-diffusion (“direct hits”) and the top 1% of diffused node scores. This matrix is converted into a diffusion-state distance (DSD) matrix [73], which was shown to improve the ability to detect gene modules on graphs [74]. Briefly, we defined a *n*-dimensional vector of the diffusion state of a node *g*_*i*_ as *u*_*i*_ = (*h*_(*i*,1)_, *h*_(*i*,2)_,⋯,*h*_(*i*,*n*)_) from each row of *H*. The DSD matrix *P* was defined by the elements *p*_(*i*,*j*)_ = ‖*u*_*i*_−*u*_*j*_‖_1_. After obtaining the DSD matrix, we converted it into a similarity matrix *S* via a Gaussian kernel defined as $S=exp({-P}^{2}{/2\sigma }_{P}^{2})$, where *σ*_*P*_ is a standard deviation of all elements of the matrix *P*. The final result is a cell type specific reweighted network for each of the 55 cell types where the weights correspond to the similarity values of the diffusion profiles of the nodes.

We also assessed the benefit of our two-step diffusion approach by comparing the node clusters obtained using a single edge diffusion from the original input node values (without node diffusion) and the clusters with node diffusion additionally. We tested this on three phenotypes and calculated silhouette index (SI), DBI, and Calinski-Harabasz-Index (CHI) to assess the goodness of the clustering (**S16 Fig**). We found that the two-step graph diffusion approach results in better clusters than the alternative of using the original input node values and then performing edge diffusion.

#### Multi-task graph clustering

To identify subnetworks in each of the cell types, we applied a multi-task graph-based clustering algorithm, MUSCARI to the 55 diffused cell type-specific networks for a given phenotype [43]. This algorithm finds graph clusters in multiple cell types by simultaneously applying spectral clustering to each cell-type-specific network while incorporating the relatedness of the cell types. A key property of this multi-task learning framework is that there is a mapping of clusters from one cell type to another. Therefore, cluster *i* in one cell type corresponds to cluster *i* in another cell type. For spectral clustering of each cell-type-specific network, we used eigenvector matrices of the regularized graph Laplacian, $L_{\tau }=I-D_{\tau }^{1/2}AD_{\tau }^{1/2}$, where *A* is the adjacency matrix and *D*_*τ*_ is a regularized diagonal matrix defined as *D*_*τ*_ = *D*+*μ*_*D*_, where *μ*_*D*_ is a mean of *D* [43].

MUSCARI takes as input the number of clusters in each cell type, input graphs and the relationship between the cell types. We ran MUSCARI with k = {6,7,8,9}, and selected k=7 based on modularity and the size of each cluster, as values of k higher than 7 tend to generate smaller clusters (**S17 Fig**). We used hierarchical clustering of H3K4me3 signal in promoters to infer the relatedness for the 55 cell types and tissues. Briefly, we created an *N* x *M* matrix of 5kb-aggregated H3K4me3 ChIP-seq signals, where *N* is the number of cell types and *M* is the total number of promoter-associated 5kb bins throughout the entire genome. We performed hierarchical clustering with Euclidean distance using unweighted average distance (UPGMA) for computing the distance between clusters. As a basis for comparison, we also clustered the Roadmap cell types by their shared 3D genome conformation. For every possible pair of Roadmap cell types, we considered the significant interactions from each cell type as a network and compared the two networks using the F-score. The F-score matrix was converted into a distance matrix based on Euclidean distance between pairs of cell types of their overall F-scores. This was used as input to hierarchical clustering. Hierarchies learned from both metrics had similar structure as assessed using the Fowlkes-Mallows index (**S8 Fig**).

#### GO enrichment on MUSCARI clusters

We performed GO enrichment on each cluster separately using a hypergeometric test with FDR correction (FDR < 0.05) and all genes in the phenotype as the background. As the number of gene lists (cluster in a cell type) we tested for enrichment was substantial (55 x 7), we applied a matrix factorization approach to identify groups of terms for groups of gene lists to enable easier interpretation. We first merged the GO terms into a matrix of -log(P) values, *X*, that was *N x M*, where *N* is the total number of terms across the 7 clusters in any cell type and *M* is all cell types in all clusters (55 x 7). On this matrix, we performed Non-negative Matrix Factorization (NMF) with k=7 and used the resulting row and column factors to cluster the terms and lists. We assessed the low-dimensional representation from NMF by computing the explained variance defined as $EV=1-\frac{var(x-\hat{x})}{var(x)}$ where *x* is the original -logP matrix and $\hat{x}$ is the reconstructed matrix. NMF produced a faithful low-dimensional representation of the enrichments explaining 94-99% of the variance. For each NMF cluster, we took the top 10 terms and the top 5 cell types/MUSCARI cluster based on the maximum values in the lower dimensional factors. We present this result in **S9 and S11 Figs**.

#### Identification of transitioning gene sets

After applying MUSCARI to all 55 cell types, we obtain cluster assignments for all genes in the network. A transitioning gene set is a gene set with a similar cluster membership in one part of the tree of cell types, but different membership, but similar across genes, in another part of the tree. We identified these gene sets based on the *de novo* clustering approach described in (Shin et al., 2021) on MUSCARI outputs [43]. Briefly, hierarchical clustering was performed on the cluster assignment profiles of genes followed by optimal leaf ordering and then gene sets were obtained with a cut height of 0.1. We removed gene sets with less than 5 genes. We interpreted these gene sets based on overlap of genes with SNPs, tendency of SNP-gene interactions to vary according to the change in cluster assignment, and Gene Ontology enrichment.

## Supporting information

S1 Fig Datasets available from the Roadmap epigenomics database across across all the cell lines and tissues in the Roadmap database (columns). Black – dataset exists in Roadmap, White –dataset absent from Roadmap.
(TIF)

S2 Fig Distribution of the number of cases in which L-HiC-Reg is better than HiC-Reg as function of training cell line (A), test cell line (B), and chromosome (C).
(TIF)

S3 Fig Individual distance-stratified correlation curves for L-HiC-Reg and HiC-Reg for chromosome 10 for all pairs of training and test cell lines from Rao et al. (TIF)

S4 Fig Fold enrichment versus P-value assessing the overlap of predicted interactions with existing ChIA-PET interactions. Shown are results for the three computational methods: L-HiC-Reg, JEME and GeneHancer.
(TIF)

S5 Fig Expression of interacting versus non-interacting genes for additional cell lines with expression data and predictions in both JEME and L-HiC-Reg. Related to Main Fig 3.
(TIF)

S6 Fig Distribution of the number of SNPs in LD with the GWAS non-coding SNPsfrom the NHGRI-EBI GWAS Catalog (left), the number of genes mapped to these SNPs (middle) and the number of pairs connect SNPs to genes across multiple cell lines (left).
(TIFF)

S7 Fig Precision (left) and recall (right) of L-HiC-Reg, JEME, GeneHancer predictions and nearest neighbor (NN) for eQTL SNP-gene associations for LD SNPs.
(TIF)

S8 Fig A. Hierarchical clustering tree showing the similarity of H3K4me3 ChIP-Seq signal in the TSS of genes. This tree was used as input for the Multi-task Graph Clustering algorithm. B.Hierarchical clustering based on the F-score of shared interactions between cell lines. C. Fowlkes-Mallows index (left) comparing the similarity of two hierarchical clusterings at different levels of clustering. D. Theassociated P-value for the observed Fowlkes-Mallows index based on random permutation.
(TIF)

S9 Fig Top Gene Ontology terms and cell lines across the 7 multi-task graph clustering clusters in Coronary Artery Disease (CAD). Rows correspond to GO term and columns correspond to a cell line, cluster ID combination. Numbers next to the cell line name represents the graph cluster. To enable extraction of meaningful annotation from our GO analysis, we applied Non-negative Matrix Factorization with seven low-dimensional factors on the -log(q-value) scores of all the GO terms across all cell lines and gene clusters. For each NMF factor, the top 10 GO terms and the top 5 cell lines-Cluster ID combinations were selected based on their values in the corresponding lower-dimensional factors.
(TIF)

S10 Fig Transitioning gene sets, which are gene sets with changes in graph cluster assignment across cell lines that exhibit concordant presence-absence of SNP-gene interactions for Coronary Artery Disease (CAD). Shown are three gene sets, #243 (A.), #196 (B.) and #200 (C.). Color heatmaps represent the cluster assignments for all 55 cell lines and the black and white heatmaps represent the presence (black) or absence (white) of the SNP-gene interaction with specific genes in the gene set across the cell lines.
(TIF)

S11 Fig Top Gene Ontology terms and cell lines across the 7 multi-task graph clustering clusters for breast cancer. Numbers next to the cell line name represents the graph cluster. To enable extraction of meaningful annotation from our GO analysis, we applied Non-negative Matrix Factorization with seven low-dimensional factors on the -log(q-value) scores of all the GO terms across all cell lines and gene clusters. For each NMF factor, the top 10 GO terms and the top 5 cell lines were selected based on their values in the corresponding lower-dimensional factors.
(TIF)

S12 Fig Transitioning gene set #225 in breast cancer. Color heatmap represents the cluster assignment across the 55 cell lines and the black and white heatmap represents the presence (black) or absence (white) of a SNP-gene interaction in a cell line.
(TIF)

S13 Fig Average area under the distance-stratified correlation curve for different training cell types predicting H1 contact counts when compared against 5kb naive hESC HiC data from Battle et al. (TIF)

S14 Fig AUROC in a Leave One Out Cross Validation (LOO-CV) setting for different values of the kernel width hyper-parameter (λ, x-axis) across several phenotypes and all 55 cell lines (y-axis). To highlight the differences across different values of λ, the mean AUROC for each row is subtracted from each row.
(TIF)

S15 Fig Selection of β for node diffusion show in the breast cancer GWAS study. (TIF)

S16 Fig Assessing the spectral clustering results with and without the node diffusion step with Silhouette index (SI), Davis Bouldin Index, and Calinski-Harabasz index (CHI) in 3 different phenotypes. Lower DBI is better, therefore we report the 1/DBI. For SI and CHI, higher is better.
(TIF)

S17 Fig Mean size of each cluster identified by multi-task graph clustering across cell lines for different phenotypes: Autism spectrum disorder, Schizophrenia, Breast cancer, Coronary artery disease and Crohn’s disease. Y-axis is the cluster ID and x-axis is the number of clusters.
(TIF)

S1 Data Genes with the top 1% highest diffused scores for breast cancer after graph diffusion for each of the cell types. A total of 1449-1821genes were recovered across the cell types.
(TXT)

S2 Data Genes with the top 1% highest diffused scores for breast cancer after graph diffusion for each of the cell types. A total of 1241-1797 genes were recovered across the cell types.
(TXT)
