# Supplementary figures and images for: Leveraging epigenomes and three-dimensional genome organization for interpreting regulatory variation
Source: PLoS Comput Biol. 2023 Jul 10;19(7):e1011286. doi: 10.1371/journal.pcbi.1011286 (PMC10358954; doi:10.1371/journal.pcbi.1011286)

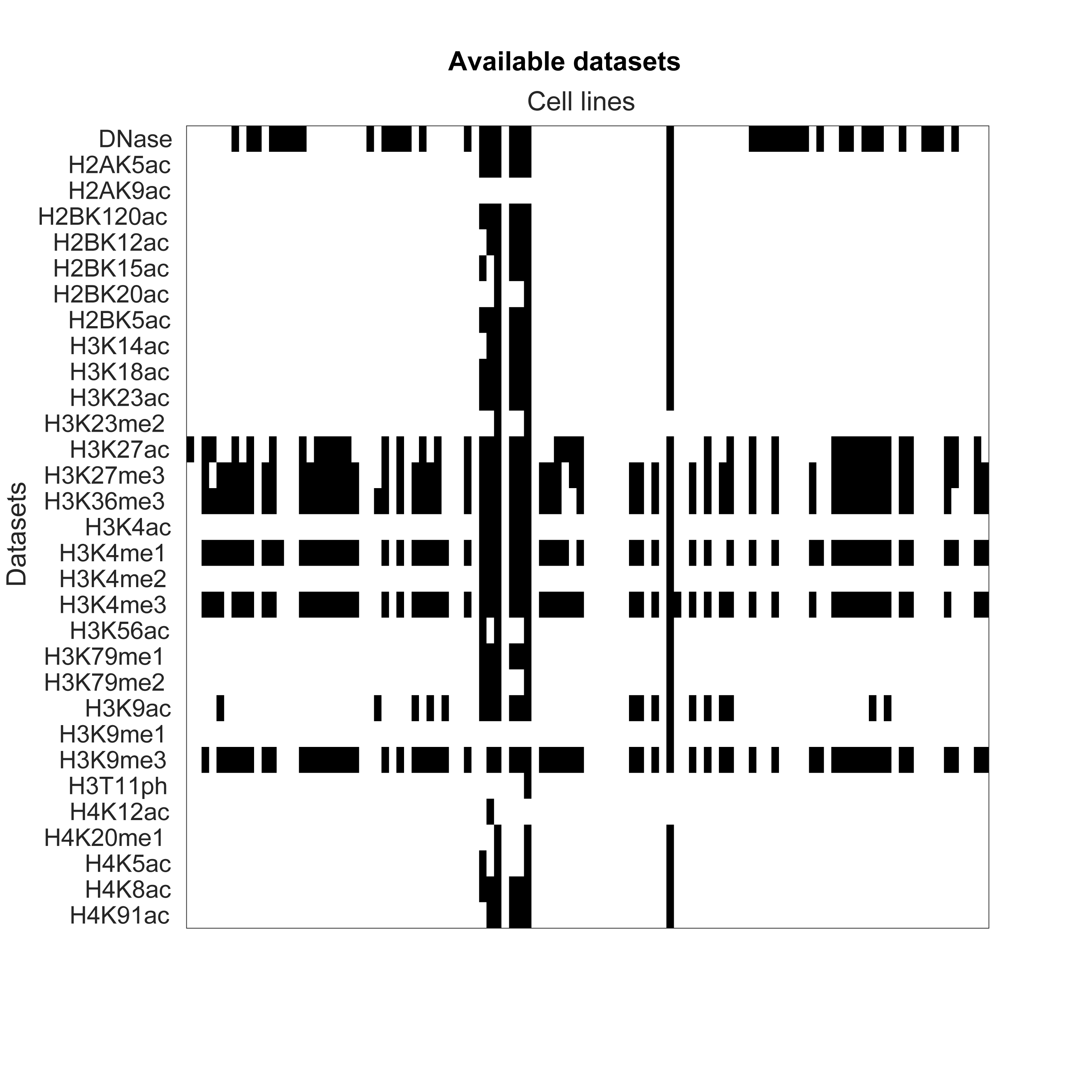

Supplement: S1 Fig — Black – dataset exists in Roadmap, White –dataset absent from Roadmap. (TIF) [file pcbi.1011286.s001.tif]

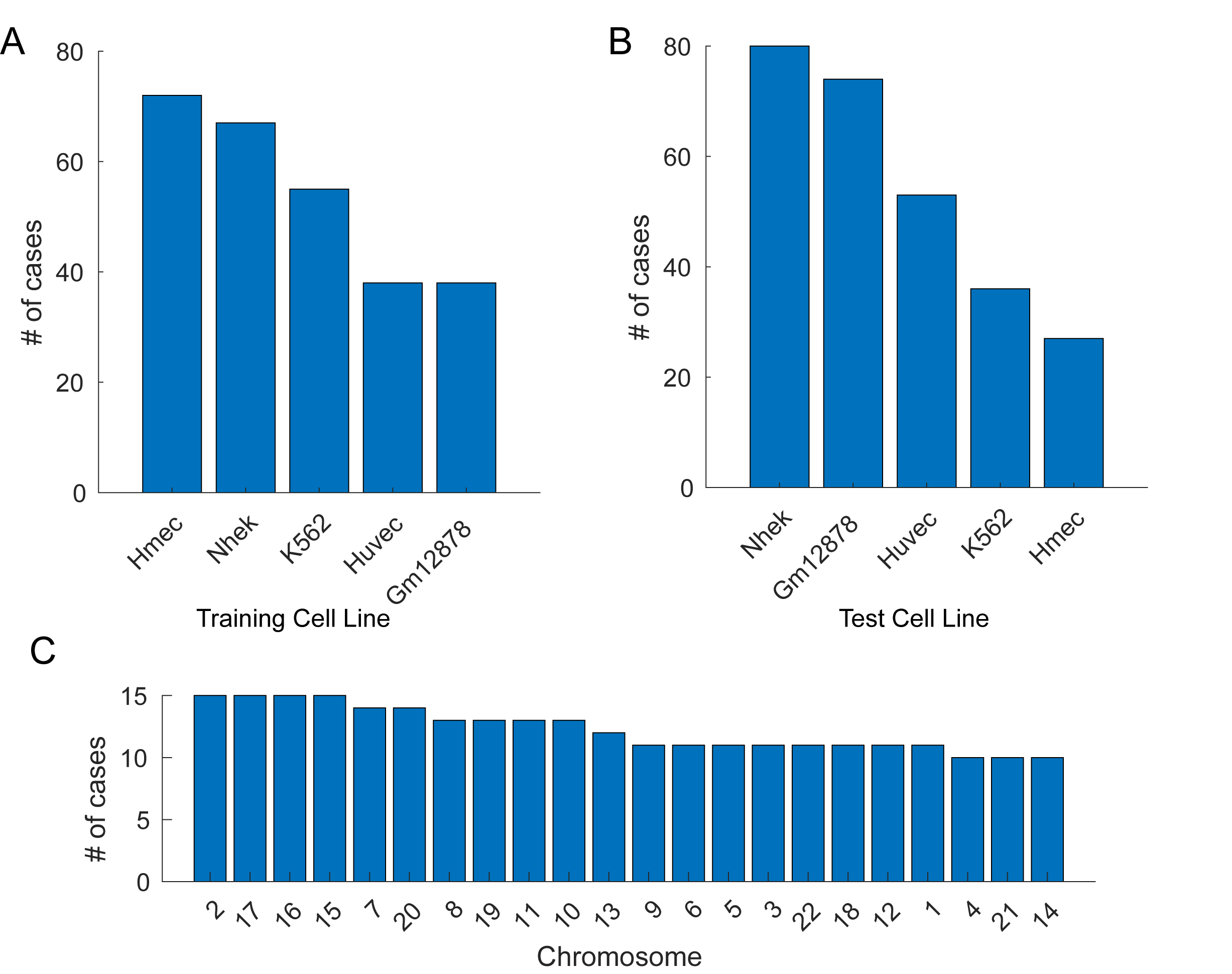

Supplement: S2 Fig — Distribution of the number of cases in which L-HiC-Reg is better than HiC-Reg as function of training cell line (A), test cell line (B), and chromosome (C). (TIF) [file pcbi.1011286.s002.tif]

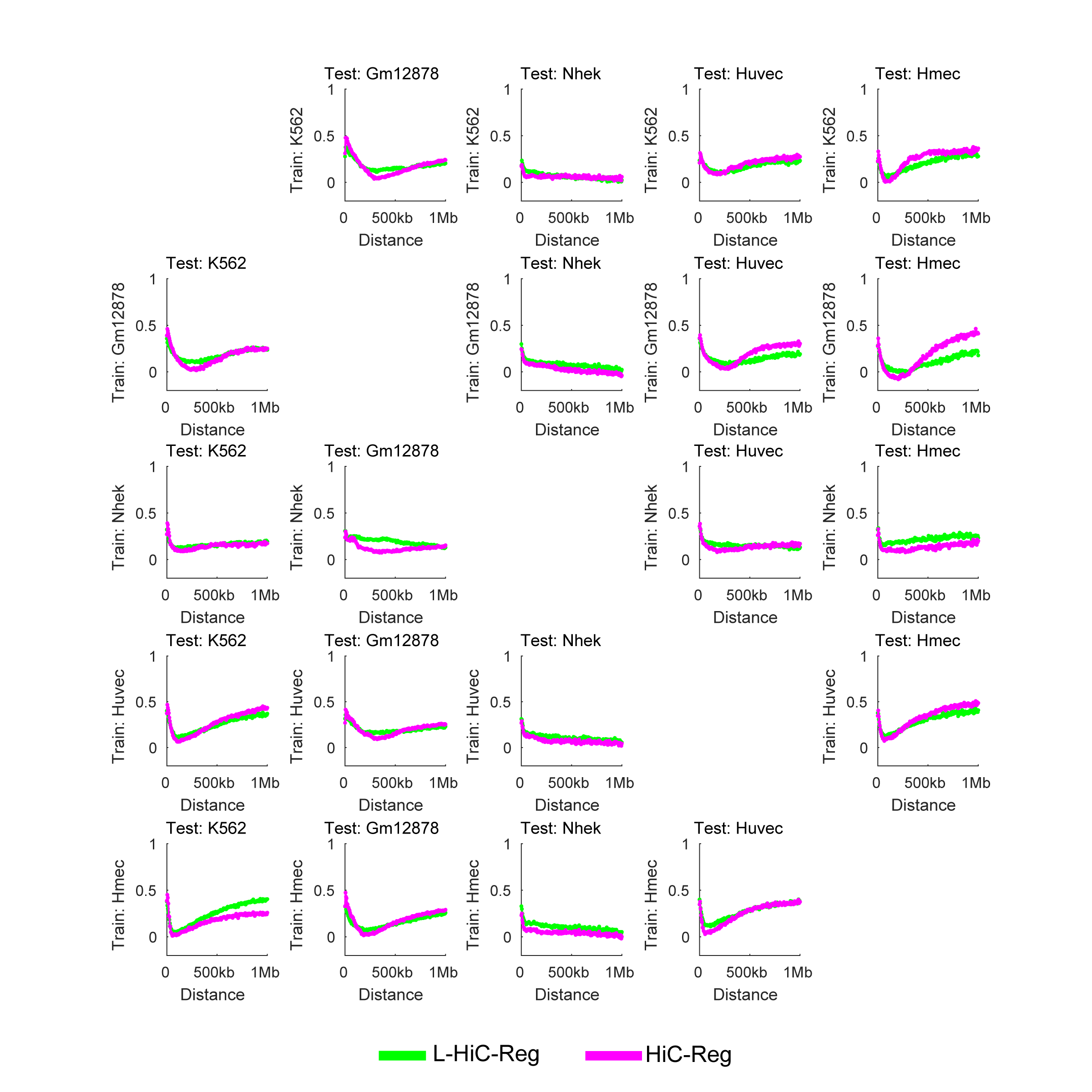

Supplement: S3 Fig — (TIF) [file pcbi.1011286.s003.tif]

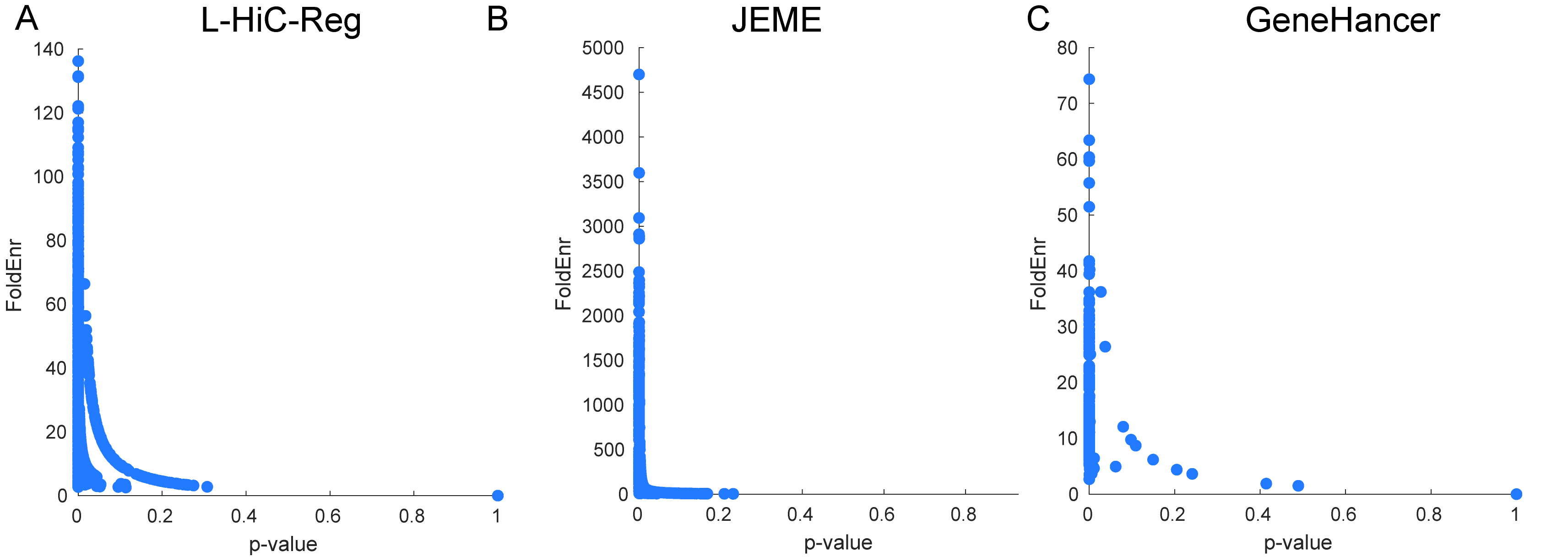

Supplement: S4 Fig — Shown are results for the three computational methods: L-HiC-Reg, JEME and GeneHancer. (TIF) [file pcbi.1011286.s004.tif]

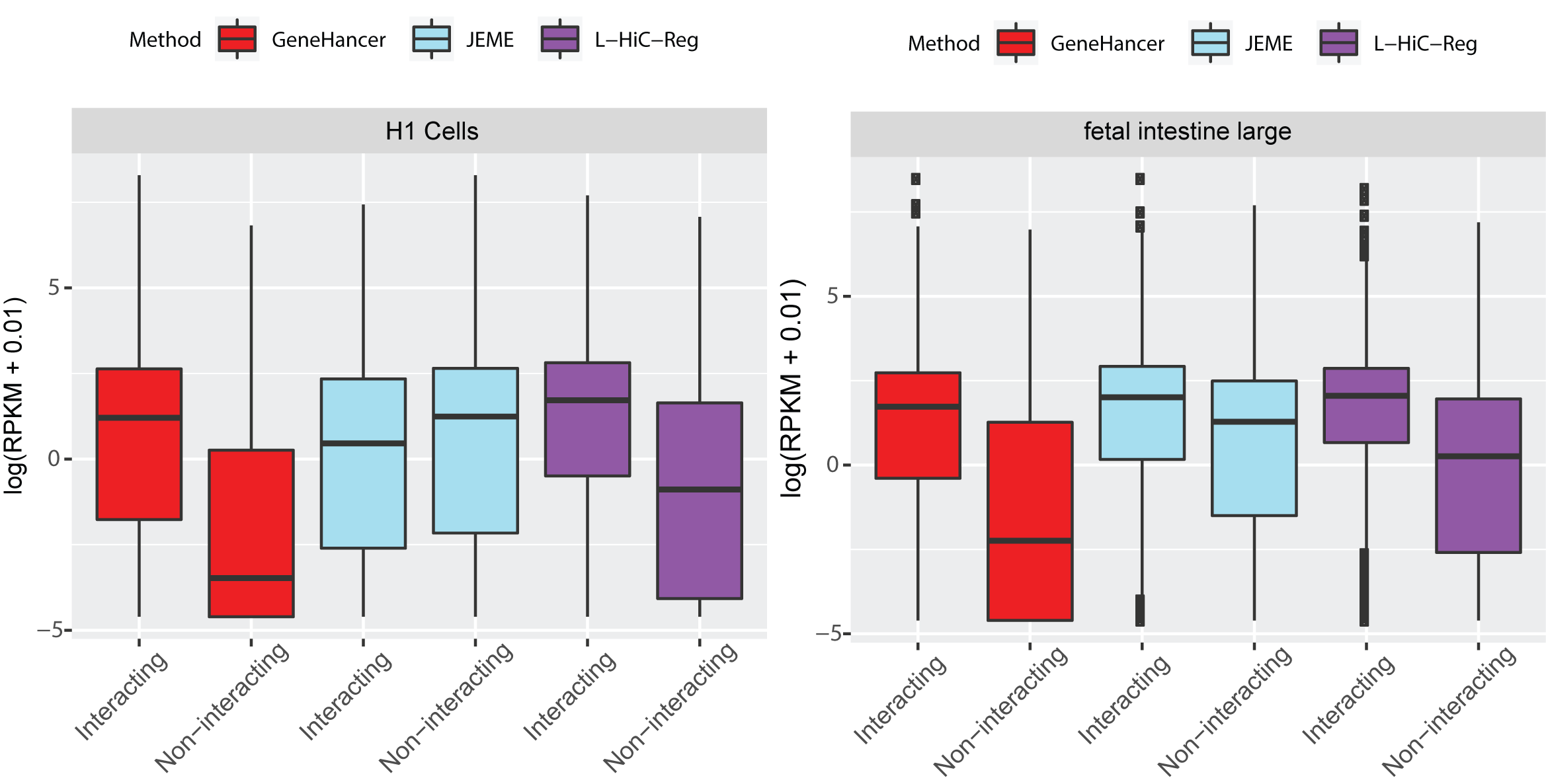

Supplement: S5 Fig — Related to Main Fig 3. (TIF) [file pcbi.1011286.s005.tif]

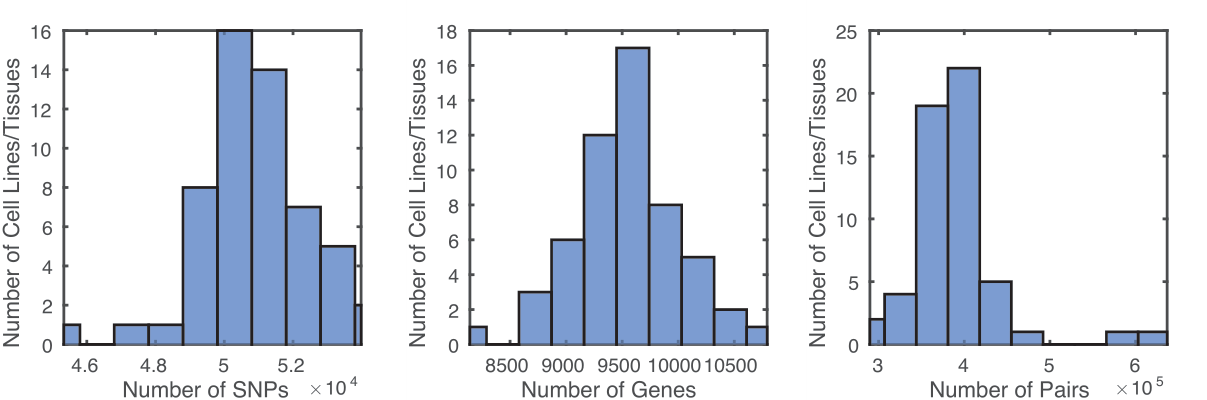

Supplement: S6 Fig — Distribution of the number of SNPs in LD with the GWAS non-coding SNPsfrom the NHGRI-EBI GWAS Catalog (left), the number of genes mapped to these SNPs (middle) and the number of pairs connect SNPs to genes across multiple cell lines (left). (TIFF) [file pcbi.1011286.s006.tiff]

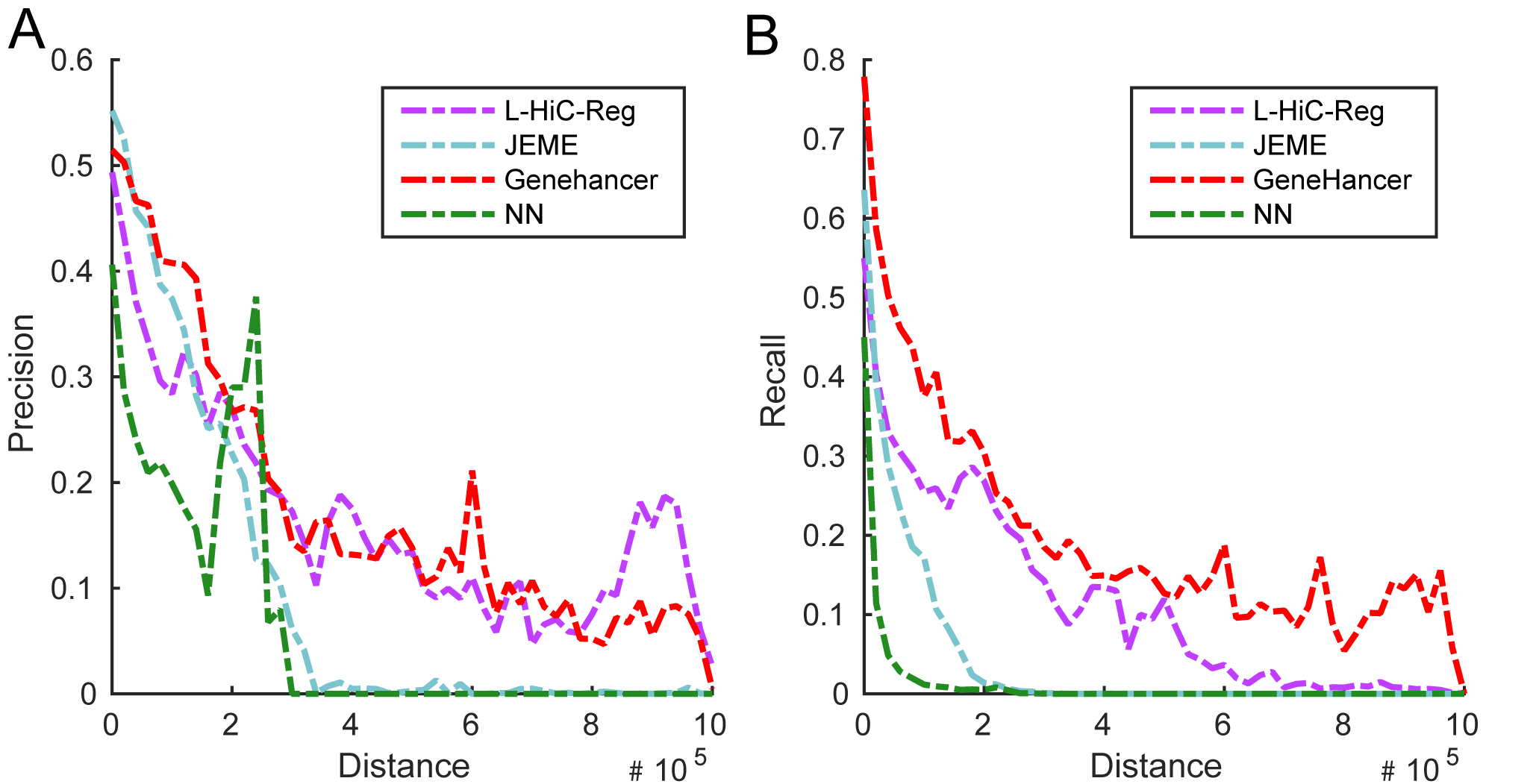

Supplement: S7 Fig — Precision (left) and recall (right) of L-HiC-Reg, JEME, GeneHancer predictions and nearest neighbor (NN) for eQTL SNP-gene associations for LD SNPs. (TIF) [file pcbi.1011286.s007.tif]

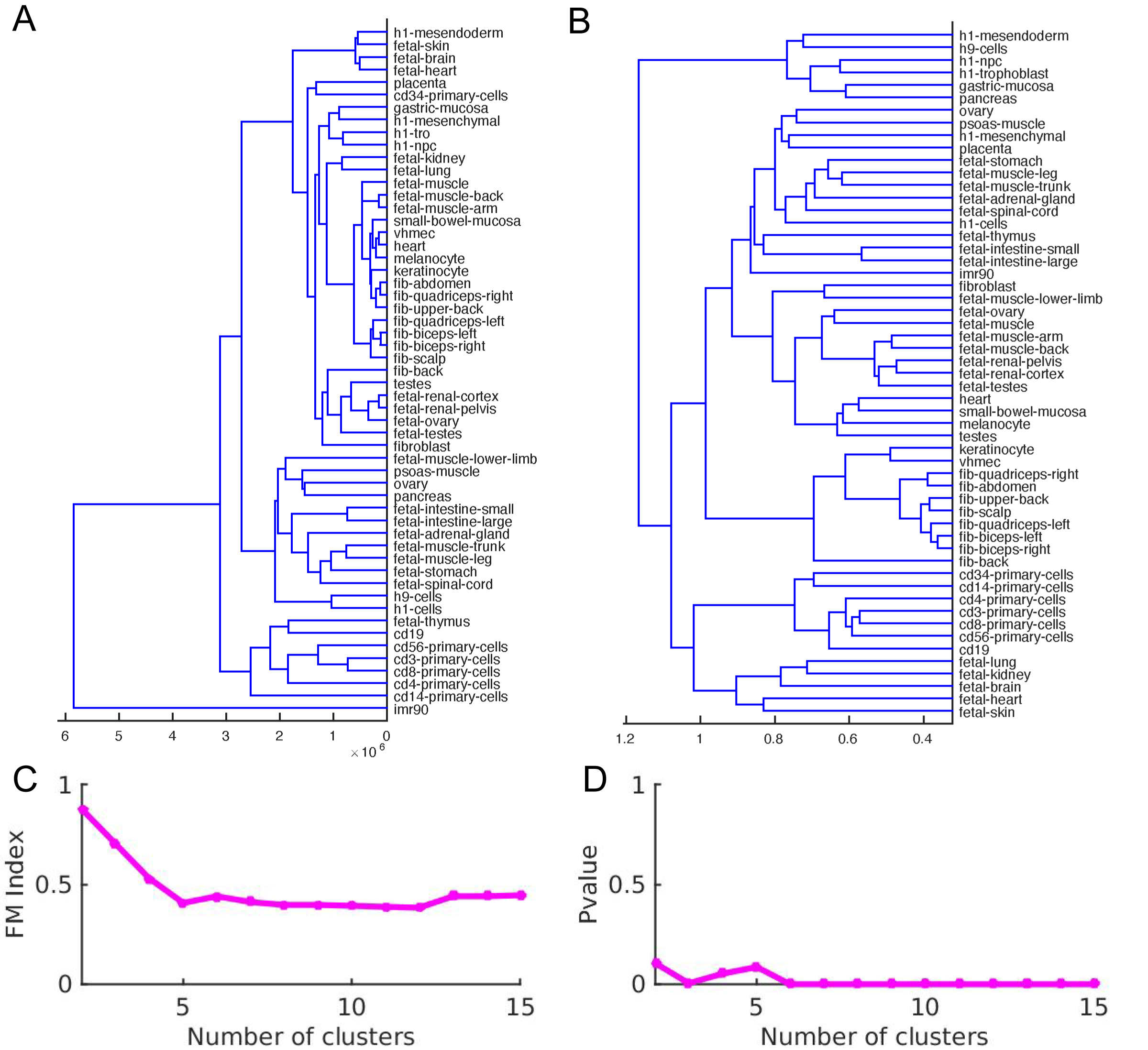

Supplement: S8 Fig — A. Hierarchical clustering tree showing the similarity of H3K4me3 ChIP-Seq signal in the TSS of genes. This tree was used as input for the Multi-task Graph Clustering algorithm. B.Hierarchical clustering based on the F-score of shared interactions between cell lines. C. Fowlkes-Mallows index (left) comparing the similarity of two hierarchical clusterings at different levels of clustering. D. Theassociated P-value for the observed Fowlkes-Mallows index based on random permutation. (TIF) [file pcbi.1011286.s008.tif]

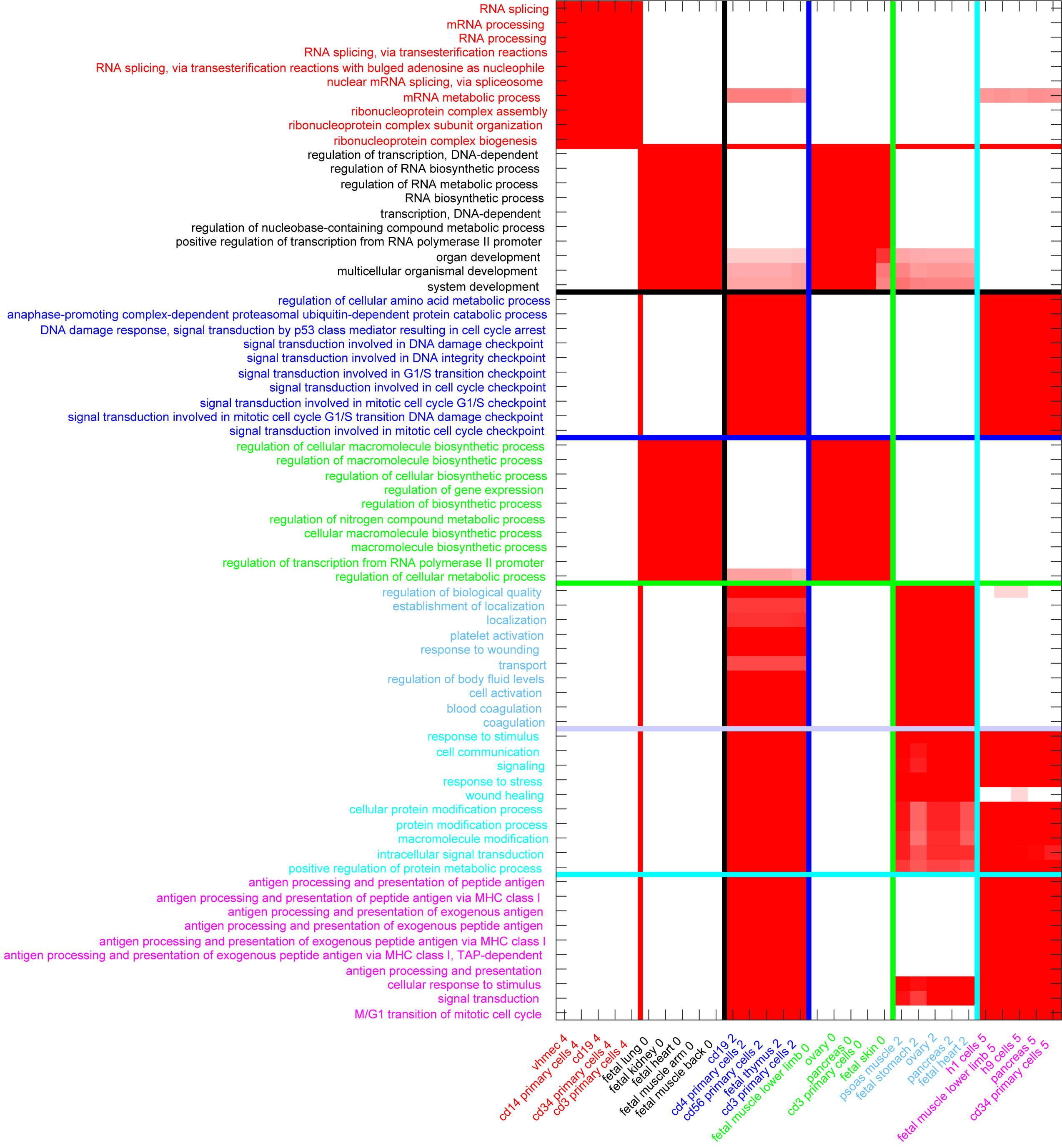

Supplement: S9 Fig — Rows correspond to GO term and columns correspond to a cell line, cluster ID combination. Numbers next to the cell line name represents the graph cluster. To enable extraction of meaningful annotation from our GO analysis, we applied Non-negative Matrix Factorization with seven low-dimensional factors on the -log(q-value) scores of all the GO terms across all cell lines and gene clusters. For each NMF factor, the top 10 GO terms and the top 5 cell lines-Cluster ID combinations were selected based on their values in the corresponding lower-dimensional factors. (TIF) [file pcbi.1011286.s009.tif]

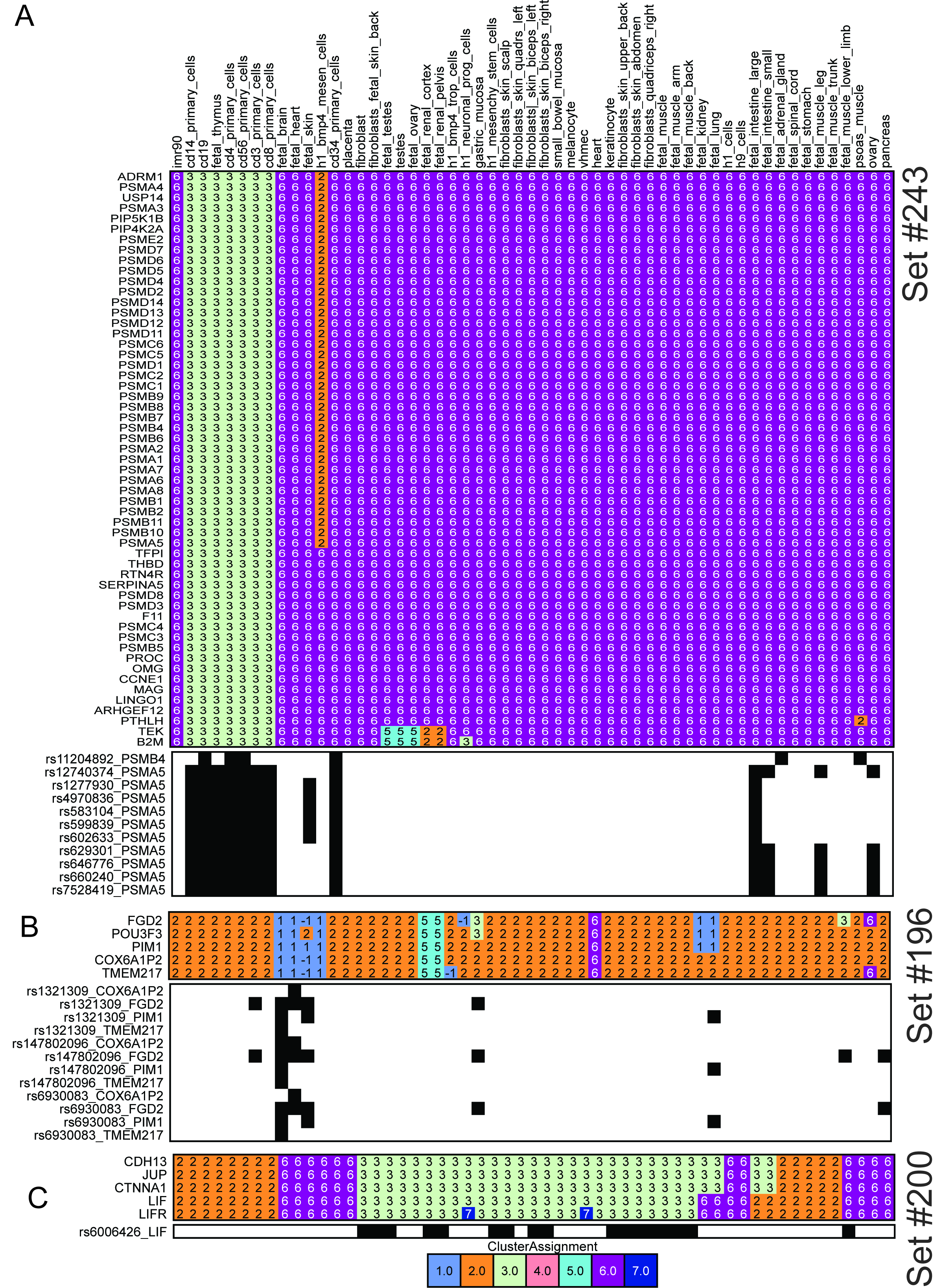

Supplement: S10 Fig — Shown are three gene sets, #243 (A.), #196 (B.) and #200 (C.). Color heatmaps represent the cluster assignments for all 55 cell lines and the black and white heatmaps represent the presence (black) or absence (white) of the SNP-gene interaction with specific genes in the gene set across the cell lines. (TIF) [file pcbi.1011286.s010.tif]

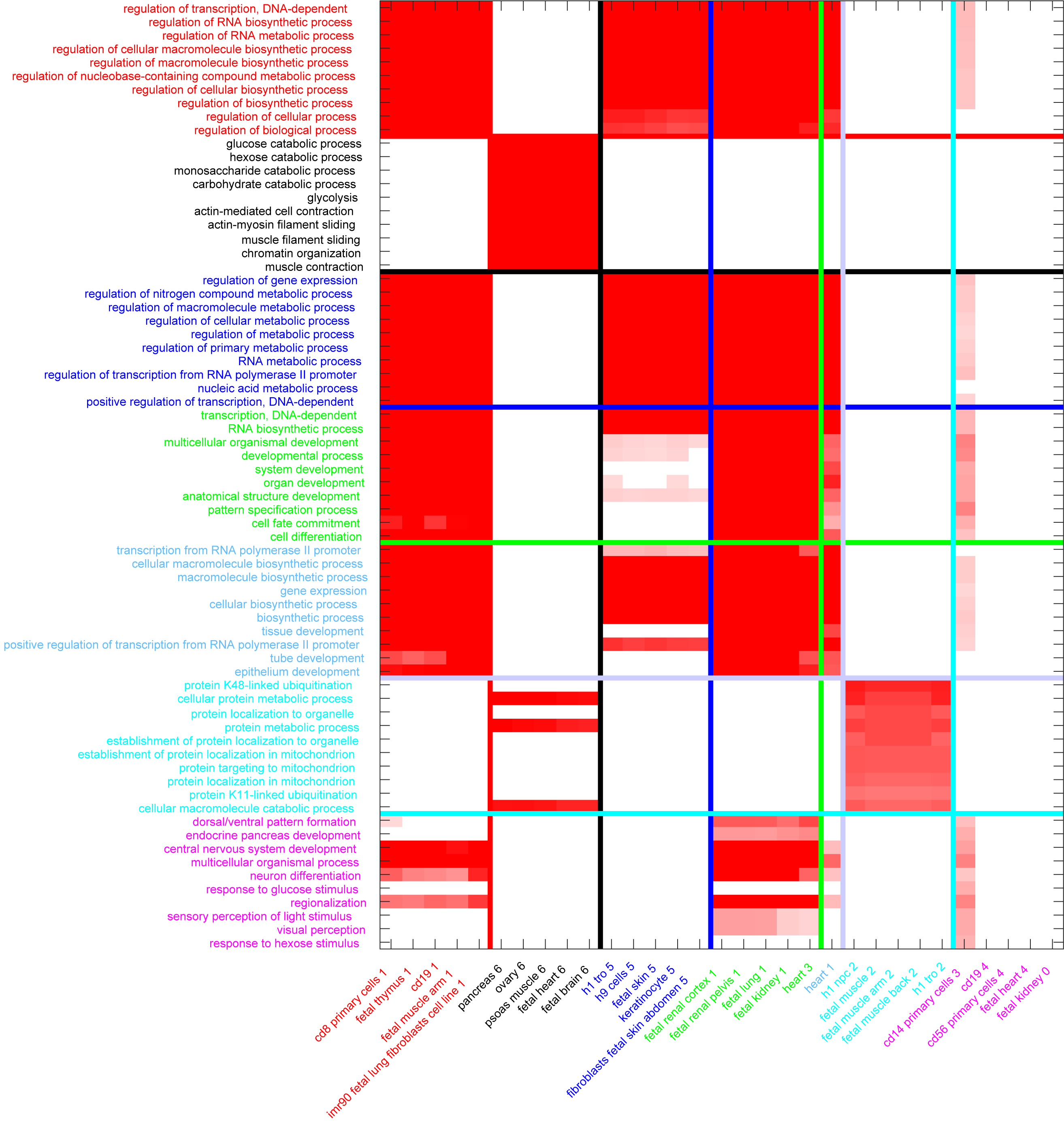

Supplement: S11 Fig — Numbers next to the cell line name represents the graph cluster. To enable extraction of meaningful annotation from our GO analysis, we applied Non-negative Matrix Factorization with seven low-dimensional factors on the -log(q-value) scores of all the GO terms across all cell lines and gene clusters. For each NMF factor, the top 10 GO terms and the top 5 cell lines were selected based on their values in the corresponding lower-dimensional factors. (TIF) [file pcbi.1011286.s011.tif]

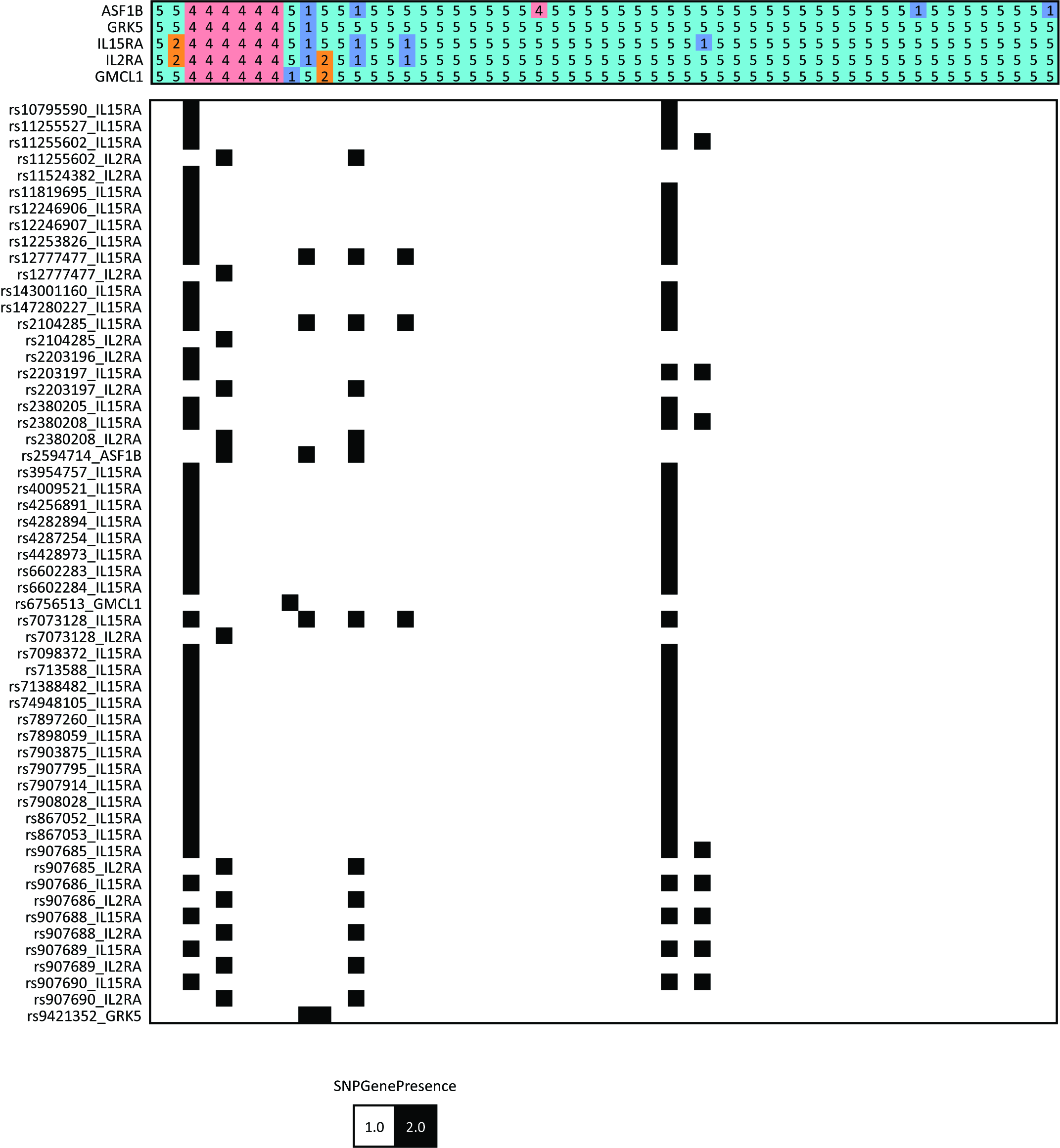

Supplement: S12 Fig — Color heatmap represents the cluster assignment across the 55 cell lines and the black and white heatmap represents the presence (black) or absence (white) of a SNP-gene interaction in a cell line. (TIF) [file pcbi.1011286.s012.tif]

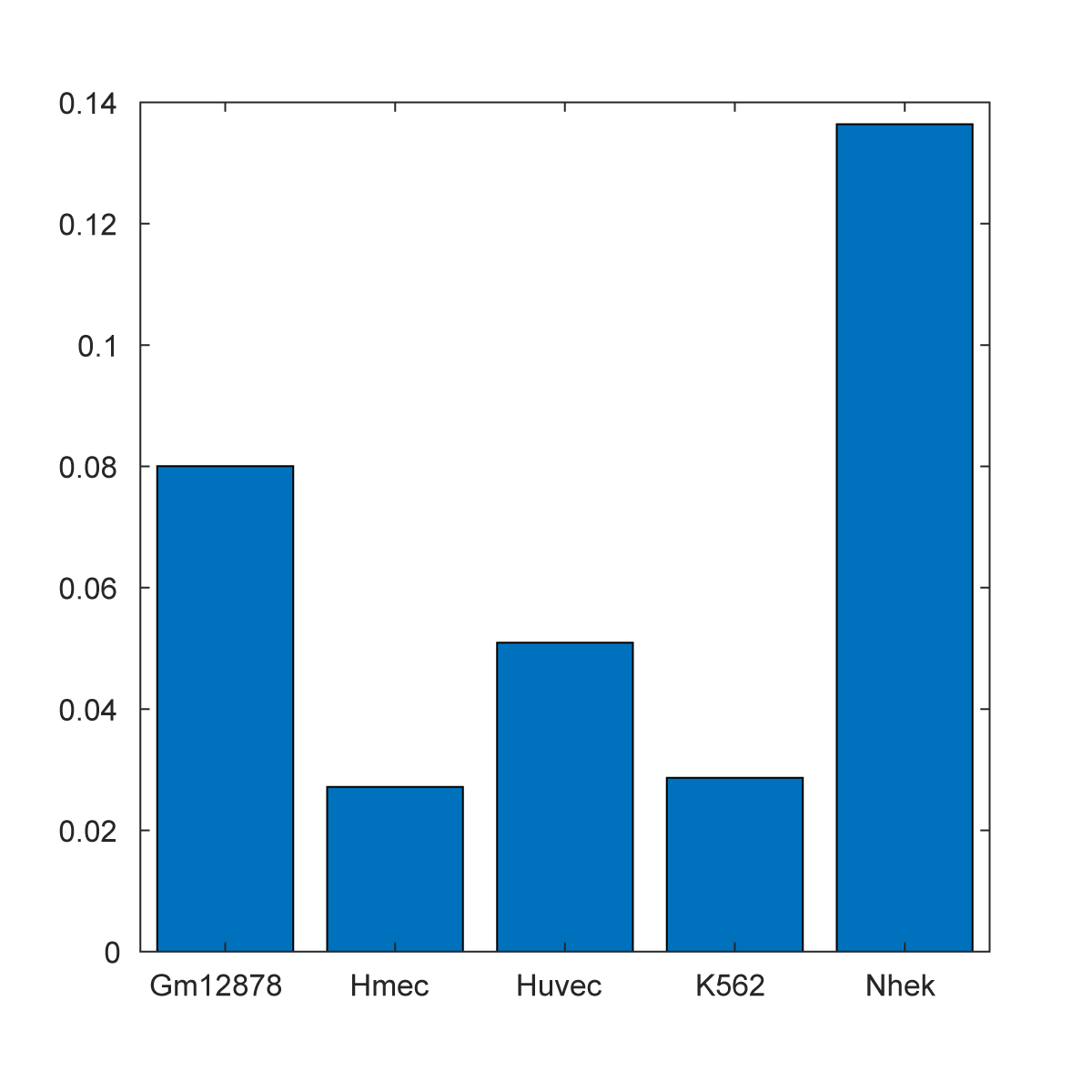

Supplement: S13 Fig — (TIF) [file pcbi.1011286.s013.tif]

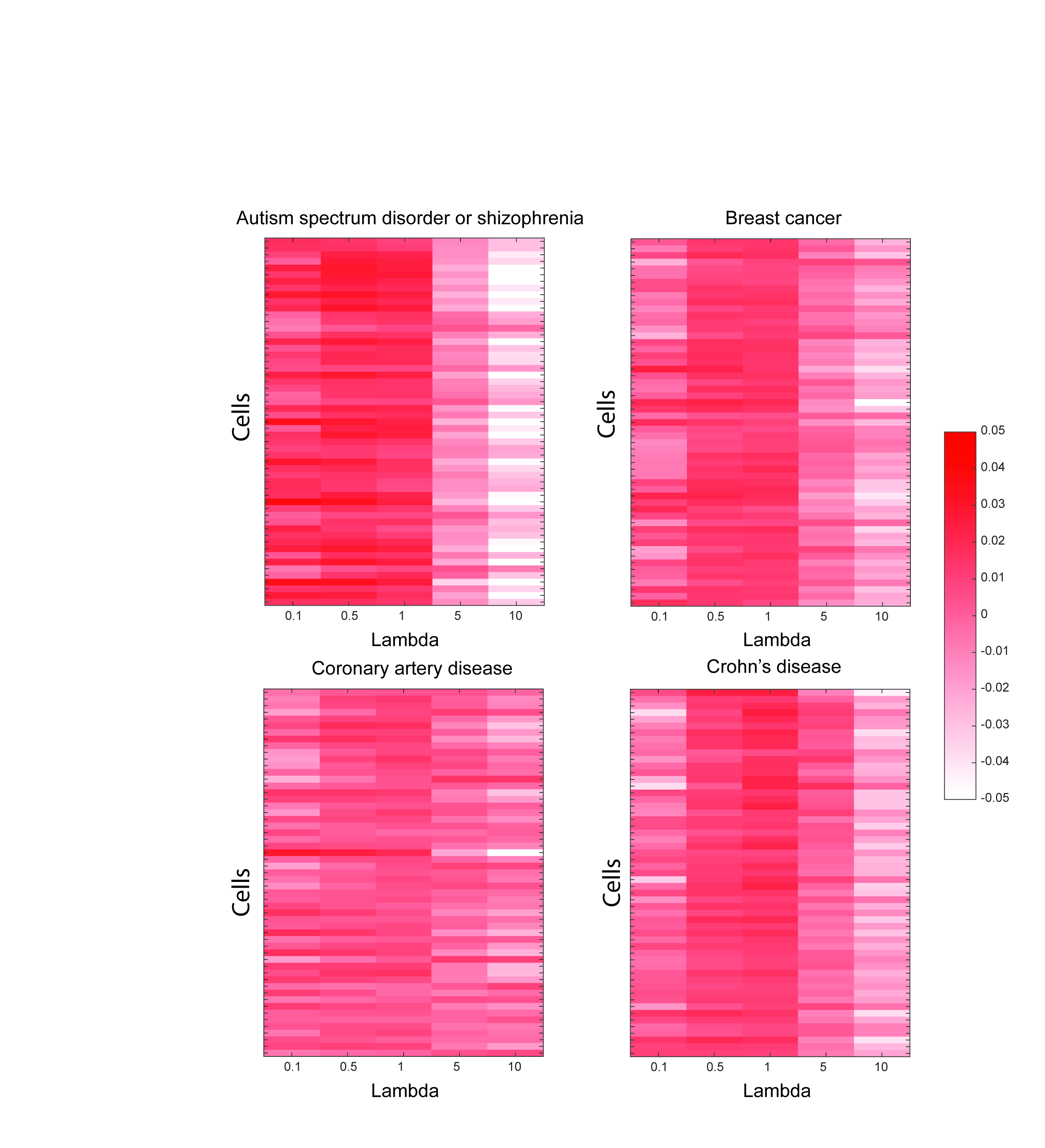

Supplement: S14 Fig — To highlight the differences across different values of λ, the mean AUROC for each row is subtracted from each row. (TIF) [file pcbi.1011286.s014.tif]

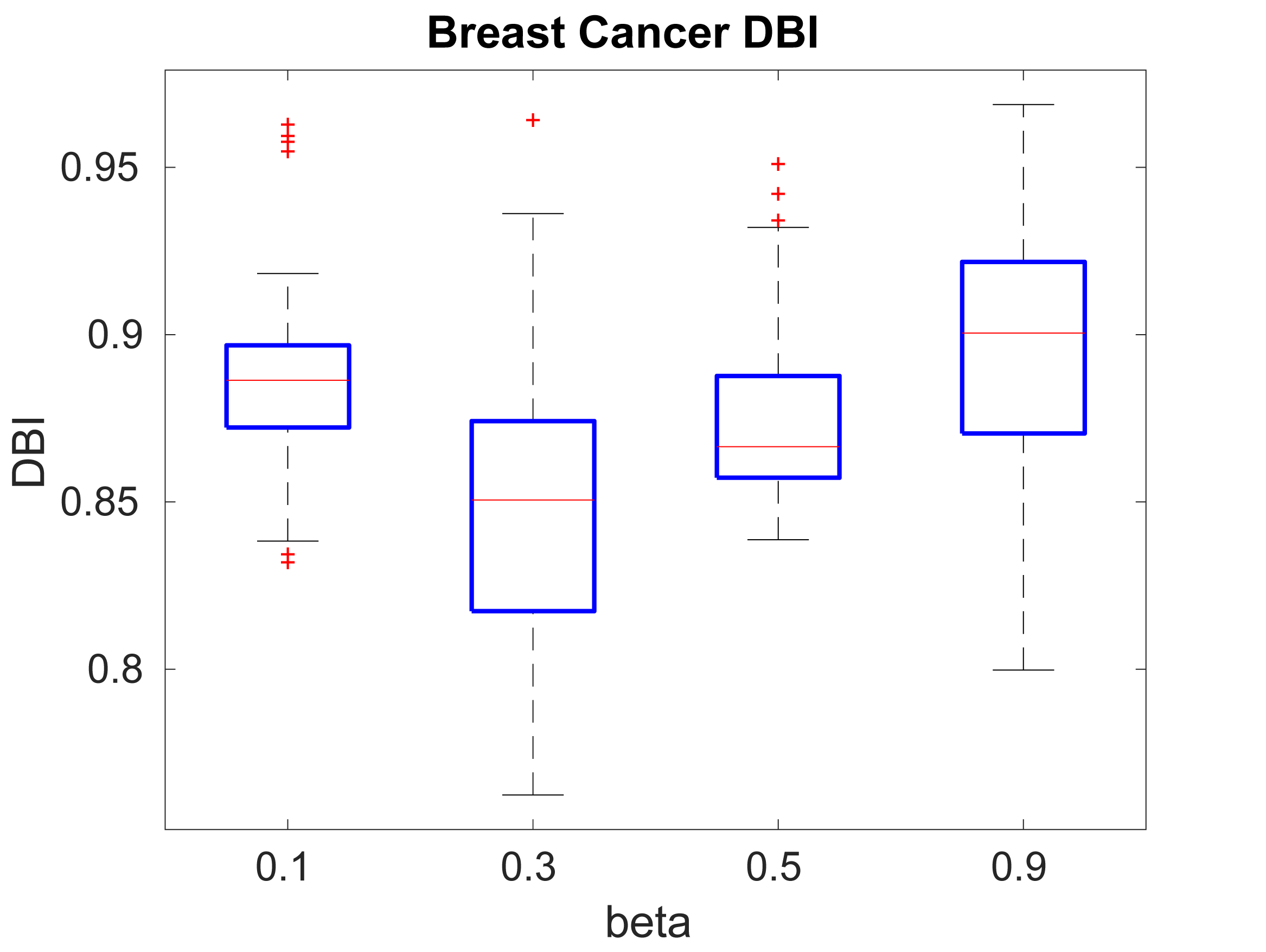

Supplement: S15 Fig — (TIF) [file pcbi.1011286.s015.tif]

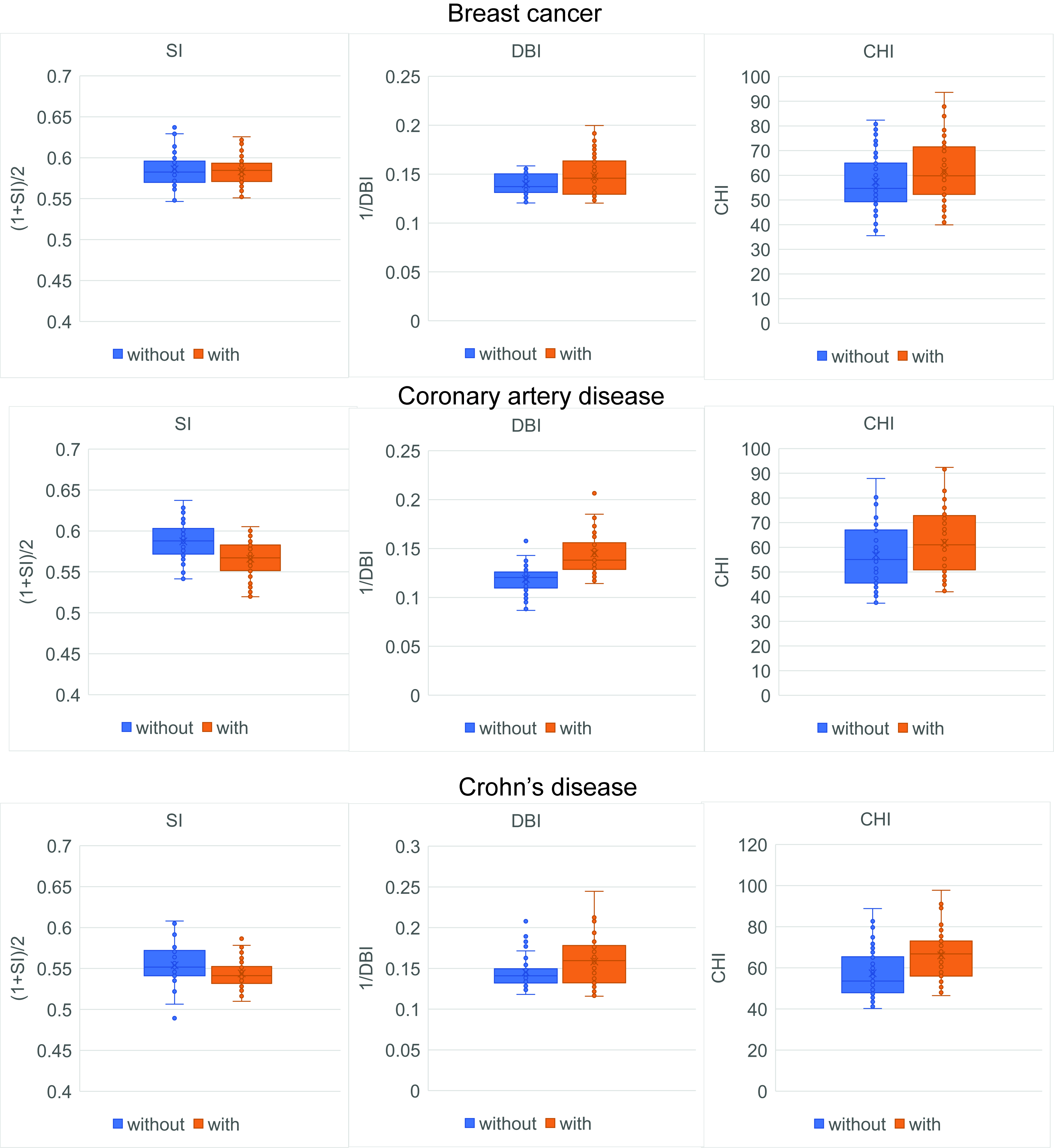

Supplement: S16 Fig — Lower DBI is better, therefore we report the 1/DBI. For SI and CHI, higher is better. (TIF) [file pcbi.1011286.s016.tif]

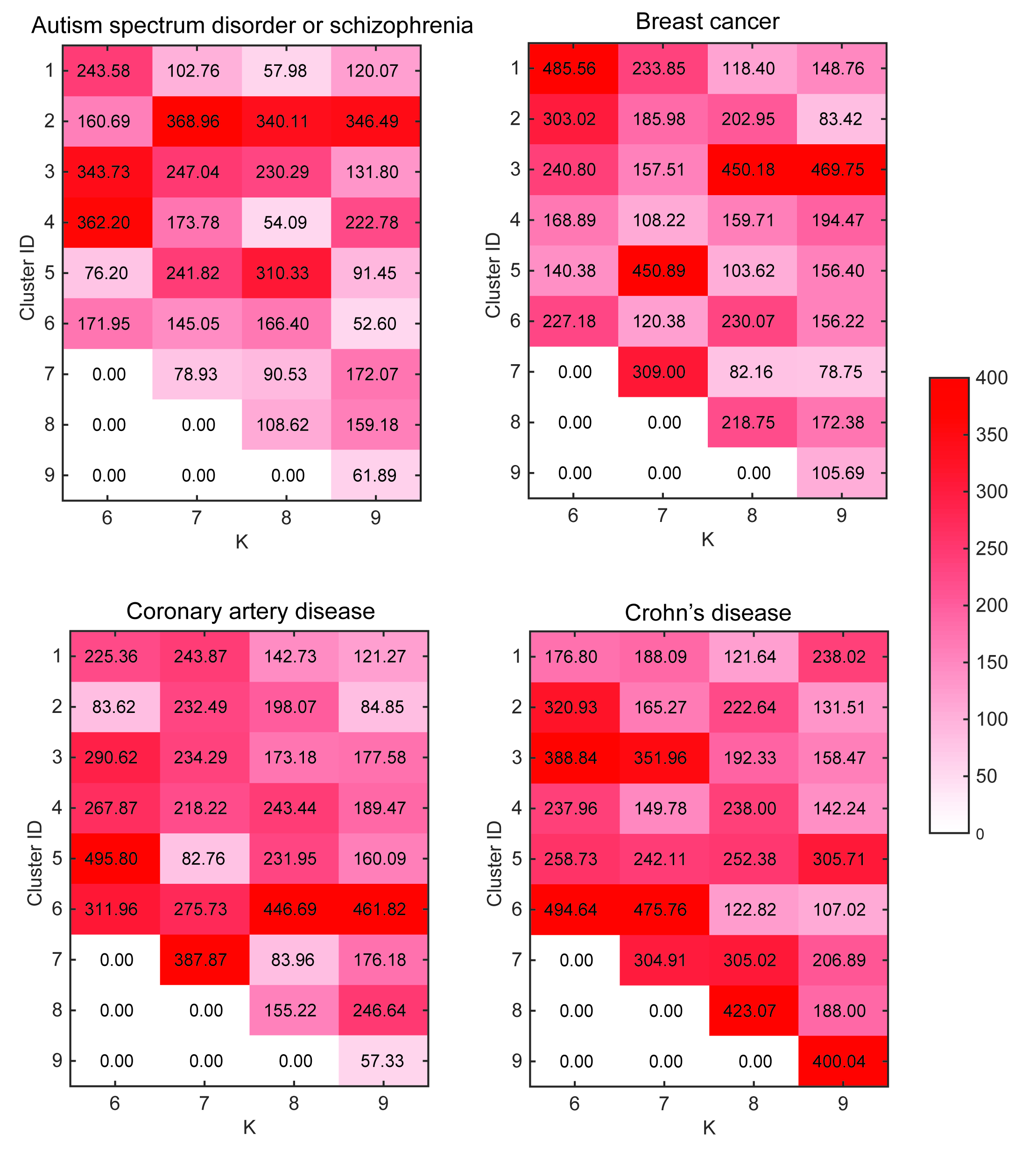

Supplement: S17 Fig — Y-axis is the cluster ID and x-axis is the number of clusters. (TIF) [file pcbi.1011286.s017.tif]
